# Disease-associated RNA and protein signatures in iPSC-derived microglia model of Alzheimer’s disease

**DOI:** 10.3389/fnins.2026.1799542

**Published:** 2026-05-26

**Authors:** Wenzhe Wu, Eun Seok Choi, Luke Liu, Veena Thamilselvan, Le Li, Meagan D. Rippee-Brooks, Kashish Khatkar, Dar-Yin Li, Denise McGrath, Aidan Manning, Sergio Barberan-Soler, Inhan Lee, Yingxin Zhao, Xiang Fang, Xiaoyong Bao

**Affiliations:** 1 Department of Pediatrics, University of Texas Medical Branch, Galveston, TX, United States; 2 Department of Computer Science, University of Rochester, Rochester, NY, United States; 3 miRcore, Ann Arbor, MI, United States; 4 Department of Mathematics, College of Natural Sciences, The University of Texas at Austin, Austin, TX, United States; 5 RealSeq Biosciences, Santa Cruz, CA, United States; 6 Department of Internal Medicine, University of Texas Medical Branch, Galveston, TX, United States; 7 Department of Neurology, University of Texas Medical Branch, Galveston, TX, United States; 8 Moody Brain Health Institute, University of Texas Medical Branch, Galveston, TX, United States; 9 Institute for Translational Science, University of Texas Medical Branch, Galveston, TX, United States; 10 Institute for Human Infections & Immunity, University of Texas Medical Branch, Galveston, TX, United States

**Keywords:** Alzheimer’s disease, iPSC-derived microglia (iMG), multiple omics, T4 PNK-sncRNA-seq, tRNA-derived RNA fragment (tRF)

## Abstract

**Introduction:**

Microglia, the resident immune cells of the central nervous system, play a critical role in maintaining neural homeostasis and regulating inflammatory responses in the brain. Increasing evidence suggests that microglial dysfunction contributes to the progression of neurodegenerative diseases, including Alzheimer’s disease (AD). However, the molecular mechanisms underlying these alterations remain incompletely understood. This study aimed to characterize disease-associated molecular changes in microglia derived from induced pluripotent stem cells (iPSCs) of sporadic AD patients and healthy donors.

**Methods:**

iPSC-derived microglia from sporadic AD patients and healthy controls were analyzed using integrated multi-omics approaches, including total RNA sequencing, proteomics, and small non-coding RNA (sncRNA) sequencing. Gene Ontology (GO) analysis was performed to identify dysregulated biological pathways from transcriptomic and proteomic datasets. In addition, a modified T4 polynucleotide kinase (T4 PNK)-based sncRNA sequencing method was used to profile disease-associated sncRNAs and identify previously uncharacterized RNA species.

**Results:**

Comparative analyses revealed significant AD-associated alterations in mRNA, protein, and sncRNA expression profiles in iPSC-derived microglia. GO analysis demonstrated dysregulation of pathways related to extracellular communication, intracellular transport, cytoskeletal organization, and protein–protein interactions. Furthermore, the modified T4 PNK–sncRNA sequencing approach identified multiple disease-associated sncRNAs, including several novel and previously uncharacterized RNA species potentially linked to AD pathology.

**Discussion:**

These findings demonstrate that iPSC-derived microglia provide a valuable model for studying molecular mechanisms associated with sporadic AD. The identified transcriptomic, proteomic, and sncRNA alterations highlight key pathways potentially involved in microglial dysfunction and neurodegeneration. In particular, the discovery of novel disease-associated sncRNAs may provide new insights into AD pathogenesis and reveal potential therapeutic targets for future investigation.

## Introduction

Alzheimer’s disease (AD) is a progressive, irreversible, and ultimately fatal neurodegenerative disorder (Dey et al., 2024; Jakob-Roetne and Jacobsen, 2009). It is marked by cognitive decline, memory loss, and changes in behavior and mood, eventually leading to complete dependence on caregivers for basic needs (Atri, 2019). The AD brain is characterized by amyloid-β (Aβ) plaques, neurofibrillary tangles, synaptic and neuronal loss, brain atrophy, neuroinflammation, disrupted neurotransmission, and impaired mitochondrial function and metabolism (Dey et al., 2024; Jakob-Roetne and Jacobsen, 2009). Despite extensive efforts to identify genetic and environmental risk factors and to elucidate the molecular mechanisms underlying disease onset and progression (Bertram and Tanzi, 2008; Kikuchi and Nakaya, 2019; Raulin et al., 2022; Stepler et al., 2022), there is currently no cure for AD.

Microglia are the resident immune cells of the central nervous system (CNS) and are critical for maintaining brain homeostasis (Crapser et al., 2021; Mehl et al., 2022). They play key roles in immune surveillance, phagocytosis, synaptic pruning, and regulation of neuroinflammation (Borst et al., 2021; Crapser et al., 2021; Mehl et al., 2022). Microglia phagocytose amyloid-β (Aβ) to facilitate Aβ clearance, engulf apoptotic cells and debris to limit inflammation, eliminate weak synapses, and modulate the extracellular matrix (ECM) to support synaptic plasticity and neural circuit stability (Cornell et al., 2022; Crapser et al., 2021; Gabandé-Rodríguez et al., 2020; Zhao et al., 2024). In response to CNS injury or diseases, microglia could become activated and release pro-inflammatory cytokines, which may have both protective and harmful effects on the brain (Gao et al., 2023; Spittau, 2017; Wendimu and Hooks, 2022). In the AD, dysfunctional microglia and dysregulated microglial activity have been shown to worsen Aβ accumulation, sustain chronic neuroinflammation, promote excessive synaptic loss, and contribute to neural degeneration. Yet, their underlying molecular mechanisms remain incompletely understood (Gabandé-Rodríguez et al., 2020; Gao et al., 2023; Hansen et al., 2018; Wang et al., 2023). In this study, we aim to identify differentially expressed genes that drive microglial dysfunction in AD, elucidate the novel mechanisms by which these genes contribute to disease progression, and ultimately reveal new therapeutic targets.

Primary human microglia are extremely difficult to obtain for experimental studies, and microglia derived from human stem cells have emerged as a valuable cell model for investigating the pathogenesis of AD (Bassil et al., 2021; Lin et al., 2018). Induced pluripotent stem cells (iPSCs) are a type of stem cell generated by reprogramming differentiated cells back into a pluripotent state. Herein, we used iPSC to first differentiate into hematopoietic progenitor cells (HPCs), and subsequently into microglia (McQuade et al., 2018). A major advantage of using iPSC-derived microglia is the ability to generate cells from both healthy individuals and AD patients, enabling direct comparison under controlled conditions. We profiled gene and protein expression in iPSC-derived microglia (iMGs) from patients with sporadic AD and healthy controls using RNA sequencing and proteomics.

Sporadic AD, also known as late-onset AD (LOAD), typically manifests after the age of 65 and is a multifactorial disease, as it arises from a complex interplay of genetic, environmental, and lifestyle factors, accounting for approximately 90%–95% of cases (Ansari et al., 2023; Reiss et al., 2022). In contrast, familial AD (fAD), which usually manifests before age 65 and is often caused by specific gene mutations in amyloid precursor protein (APP), presenilin 1 (PSEN1), and presenilin 2 (PSEN2), accounts for only 5%–10% of AD cases (Wu et al., 2012). In this study, we focused on sporadic AD-altered gene/protein/sncRNA expression in iMG.

Small noncoding RNAs (sncRNAs) are potent regulators of numerous biological processes, and their dysregulation has been implicated in several diseases, including AD (Alexandrov et al., 2012; Jain et al., 2019; Salta and De Strooper, 2012; Watson et al., 2019). Multiple groups, including ours, have reported AD-altered brain sncRNA profiles, notably involving recently characterized tRNA-derived fragments (tRFs) (Alexandrov et al., 2012; Jain et al., 2019; Wu W. et al., 2019; Wu et al., 2021). These tRFs are generated from a limited set of mature tRNAs and function not only as potential biomarkers with high disease specificity but also as regulators of neuron functions (Wu W. et al., 2019; Wu et al., 2021). Additionally, we performed transcriptomic and proteomic profiling of iMGs from AD patients and healthy controls. This multi-omics analysis revealed widespread microglial dysregulation, particularly in pathways involving extracellular communication, intracellular transport, cytoskeletal organization, and protein–protein interactions.

## Materials and methods

### iPSCs culture

Induced pluripotent stem cells (Catalog #s: AG27605, AG27607, AG27609, AG25367, AG27602, AG28262, and AG27611) were obtained from the Coriell Institute and cultured in mTeSR Plus medium (Cat. # 100-0276, STEMCELL Technologies, Vancouver, BC) on Matrigel-coated 6-well plates. Matrigel was purchased from Corning via Fisher Scientific (Cat. # CB-40234A, Waltham, MA).

### Differentiation of iPSCs to hematopoietic progenitor cells (HPCs)

Induced pluripotent stem cells were differentiated into HPCs using the STEMdiff Hematopoietic Kit (Cat. # 05310, STEMCELL Technologies) following the manufacturer’s instructions. Briefly, iPSCs were seeded on Matrigel-coated 12-well plates in mTeSR Plus medium and allowed to form ∼16–40 colonies per well overnight. On Day 0, the medium was replaced with STEMdiff Hematopoietic Medium A from the STEMdiff Hematopoietic Kit, with subsequent half-medium changes as directed. On Day 3, cultures were switched to Medium B, again with half-medium changes. Non-adherent cells harvested on Day 12 were confirmed as HPCs and used immediately for microglial differentiation.

### Differentiation of HPCs to Microglia

Hematopoietic progenitor cells were further differentiated using the STEMdiff Microglia Differentiation Kit (Cat. #: 100-0019, STEMCELL Technologies) and matured with the STEMdiff Microglia Maturation Kit (Cat. #: 100-0020, STEMCELL Technologies), per the manufacturer’s protocol. On Day 0, 2 × 10^5^ HPCs were plated in one Matrigel-coated 6-well plate containing 2 mL Microglia Differentiation Medium, with 1 mL fresh medium added every other day. On Day 12, cells were collected by centrifugation, resuspended in 2 mL fresh Differentiation Medium, and replated onto Matrigel-coated 6-well plates; medium was topped up every other day. On Day 24, cells were again collected, resuspended in Microglia Maturation Medium, and seeded onto Matrigel-coated 6-well plates. They were then fed every other day for an additional 6 days to complete maturation.

### Immunofluorescence (IF) staining

After 6 days of maturation, iMGs were seeded onto 48-well plates, pre-coated with fibronectin (Sigma-Aldrich, St. Louis, MO). The following day, cells were washed three times with PBS and fixed with 4% paraformaldehyde (PFA) for 20 min at room temperature (RT). Cells were then permeabilized with 0.1% Triton X-100 for 10 min at RT, followed by three additional PBS washes. Before staining, the cells were treated with a blocking buffer composed of PBS with 2% BSA and 0.1% Tween-20 for 1 h. Primary antibodies against CD45 (Cat#: 368508, BioLegend, San Diego, CA) and P2RY12 (Cat#: 392103, BioLegend), two biomarkers of microglia, were diluted in blocking solution and incubated with the cells overnight at 4 °C. After washing three times with PBS, nuclei were counterstained with DAPI (Cat#: 62248, Fisher Scientific) for 5 min at RT. Images were acquired using a KEYENCE BZ-X800 series all-in-one fluorescence microscope.

Phagocytosis assay

To prepare fibrillar fluorescent amyloid-β 1-42 (Aβ1-42), HiLyte Fluor 555-labeled β-amyloid peptide 1-42 (AnaSpec, Fremont, CA) was first reconstituted in 0.1% NH4OH at a concentration of 10 mg/mL. The solution was then diluted to 1 mg/mL with PBS and incubated at 37 °C for 48 h to allow fibril formation (Nabi et al., 2025). Before use, the fibrillar Aβ1-42 was thoroughly mixed to ensure uniform dispersion.

For phagocytosis assays, floating iMGs were seeded onto Matrigel-coated 48-well plates at a density of 3 × 104 cells/cm^2^ in STEMdiff Microglia Maturation Medium. Cells were then incubated with 15 μg/mL of fibrillar Aβ1-42 for 2 h at 37 °C to monitor the uptake as described (Prakash et al., 2021). The uptake of fibrillar Aβ1-42 was assessed by fluorescent images captured using a KEYENCE BZ-X800 series all-in-one fluorescence microscope (Osaka, Japan).

### RNA and protein sample preparation

After 6 days of maturation, iMG cells were harvested, and RNA was extracted using TRIzol^®^ Reagent with the PureLink^®^ RNA Mini Kit (Cat#: 12183025, Fisher Scientific), according to the manufacturer’s instructions. In brief, after adding chloroform and phase separation, the aqueous phase containing RNA was transferred to the Spin Cartridge provided in the kit. The remaining interphase and organic phase were then processed for protein extraction following the TRIzol^®^ manufacturer’s protocol.

### RNA-seq

RNA samples were submitted to RealSeq Biosciences (Santa Cruz, CA) for library preparation and RNA sequencing. Libraries were prepared using the Zymo-Seq RiboFree Total RNA Library Kit with 8 μL of total RNA input and 13 PCR cycles. Libraries were pooled at equal concentrations and profiled using a DNA Tapestation and dsDNA High Sensitivity Qubit assay before sequencing on the Singular G4 platform. Sequencing was performed to generate 2 × 150 bp paired-end reads. All datasets were normalized using the DESeq2 scaling method. Differentially expressed genes were identified based on a *p*-value < 0.05 and a log_2_ fold change > 0.8.

### T4 PNK-sncRNA-seq

In this study, we further examined sncRNA expression in iMGs from sporadic AD and healthy controls, using T4 PNK-sncRNA-seq, a modified sequencing method designed to overcome barcode ligation biases inherent in standard library preparations. Many sncRNAs, including tRFs, lack 3’-hydroxyl ends required for efficient barcode ligation, leading to their underrepresentation in conventional methods (Wu et al., 2022). Therefore, before sncRNA seq, we pretreated RNAs with T4-PNK to make sncRNAs homogeneously with 3’-hydroxyl ends. In brief, 15 μL of RNAs was pretreated with 10 units of T4 PNK (New England Biolabs, Ipswich, MA) in a final reaction volume of 50 μL. After incubating them at 37 °C for 30 min, followed by heat inactivation at 65 °C for 20 min, the treated RNAs were purified into 15 μL of nuclease-free water using the Zymo RNA Clean and Concentrator-5 kit (Cat#: 50-444-565, Zymo Research, Irvine, CA), following the small RNA enrichment protocol according to the manufacturer’s instructions. RealSeq-Biofluids libraries were then prepared using 10 μL of treated RNA input and 20 PCR cycles. Libraries were pooled at equal concentrations and profiled using a DNA Tapestation and dsDNA High Sensitivity Qubit assay before sequencing on the NextSeq 550 platform. Sequencing was performed using single-end 75 bp reads.

To analyze the seq data, adaptor sequences were first removed using Cutadapt and reads with a length of more than 15 bp were extracted. We further filtered out RNAs with counts <10 and all rRNA sequences, using the remaining reads as the cleaned input. In terms of the mapping databases, we prepared tRF5 and tRF3 databases using the identical sequences derived from different tRNAs (sequences downloaded from tRNA genes using the Table Browser of the UCSC genome browser). We also prepared tRF1 sequences using the genome locations of tRNAs. Our in-house small RNA database includes (1) these tRFs, (2) miR/snoR sequences downloaded from the UCSC genome browser, and (3) piRNA sequences downloaded from piRBase.^1^ The cleaned input reads were mapped to our in-house small RNA database using bowtie2 (v2.4.1), allowing two mismatches (option N-1). After we mapped the cleaned input reads to the small RNA database, the unmapped sequences were then mapped to the hg38 genome using the bowtie2 pre-built index (GRCh38_noalt_as) to detect all human sequences.

Raw read counts were normalized with the DEseq2 median of ratios method. Differentially expressed genes were determined by *p*-value < 0.05, fold change > 2, and mean of normalized counts > 10 in either CN or AD group. Unsupervised hierarchical clustering was performed using the Pearson correlation coefficient.

### Proteomics

Trypsin digestion was performed as previously described (Zhao et al., 2021, 2022). Proteins were dissolved in 8 M guanidine and reduced with 10 mM DTT for 30 min, followed by alkylation with 20 mM iodoacetamide (IAA) for 1 h in the dark. An aliquot containing 10 μg of protein was digested overnight with a Lys-C/trypsin mix. The resulting peptides were desalted using C18 TopTips (Pierce, Rockford, IL).

For LC-MS/MS analysis, a nanoflow ultra-high performance liquid chromatography (UHPLC) instrument (Easy nLC, Fisher Scientific) was coupled online to a Q Exactive mass spectrometer (Fisher Scientific) with a Nano electrospray ion source (Fisher Scientific). Peptides were loaded onto a C18-reversed phase column (25 cm long, 75 μm inner diameter) and separated with a linear gradient of 5%–35% buffer B (100% acetonitrile in 0.1% formic acid) at a flow rate of 300 nL/min over 120 min. Each sample was analyzed by LC-MS/MS twice. MS data were acquired using a data-dependent Top10 method, dynamically choosing the most abundant precursor ions from the survey scan (350–1,400 m/z) using HCD fragmentation. Survey scans were acquired at a resolution of 70,000 at m/z 400. Unassigned precursor ion charge states, as well as singly charged species, were excluded from fragmentation. The isolation window was set to 3 Da and fragmented with normalized collision energies of 28. The maximum ion injection times for the survey scan and the MS/MS scans were 20 and 120 ms, respectively, and the ion target values were set to 3E6 and 1e5, respectively. Selected sequenced ions were dynamically excluded for 30 s. Data was acquired using Xcalibur software (Fisher Scientific).

Raw mass spectrometry (MS) data were analyzed using MaxQuant software (version 1.5.2.8) with the Andromeda search engine (Cox and Mann, 2008; Cox et al., 2014). The initial maximum allowed mass deviation was set to 10 ppm for monoisotopic precursor ions and 20 ppm for MS/MS peaks. Enzyme specificity was set to trypsin, defined as cleavage C-terminal to arginine and lysine residues (excluding proline), allowing up to two missed cleavages. Spectra were searched against the SWISSPROT human protein database (42,130 human protein entries), supplemented with 248 common contaminants, and concatenated with reversed sequences to estimate false discovery rates. Protein identification requires at least one unique or razor peptide per protein group. Quantification was performed using MaxQuant’s built-in extracted ion chromatogram (XIC)-based label-free quantification (LFQ) algorithm, *MaxLFQ* (Cox et al., 2014). A 1% false discovery rate (FDR) was applied at both the peptide and protein levels. The minimum required peptide length was set to 8 amino acids. For downstream statistical analysis, MaxQuant output was processed using the Perseus platform (version 1.5.5.3) (Tyanova et al., 2016). Contaminants, reverse hits, and proteins identified only by site were excluded. LFQ intensity values were log_2_-transformed, and proteins with fewer than three valid LFQ values were filtered out. The remaining missing values were imputed from a normal distribution (width = 0.3; downshift = 1.8).

### qRT-PCR

To confirm the RNA seq and proteomics results, RNAs were subjected to reverse transcription into cDNA using iScript™ cDNA Synthesis Kit (Bio-Rad, Hercules, CA) according to the manufacturer’s instructions. qRT-PCR was performed using iTaq Universal SYBR Green Supermix (Bio-Rad) with primers specific to the interested genes in the CFX Connect Real-Time PCR System (Bio-Rad, Hercules, CA, United States). The information on primers for qRT-PCR is shown in Table 1.

**TABLE 1:** Primers for qRT PCR.

Gene name	Primers	Sequence (5′–>3′)
HLA-DRA	F	AGC TGT GGA CAA AGC CAA CCT G
	R	CTC TCA GTT CCA CAG GGC TGT T
AIF1	F	CCC TCC AAA CTG GAA GGC TTC A
	R	CTT TAG CTC TAG GTG AGT CTT GG
NANS	F	TGG ACG TAG CCA AGC GCA TGA T
	R	GCC TCT CCA AGG CTT TCC GAT T
NPC2	F	GGA GTG GCA ACT TCA GGA TGA C
	R	CTG GAG GTG CTG TCA AGA GTC T
ACTN1	F	CAG GAC CGT GTG GAG CAG ATT G
	R	CAG ATT GTC CCA CTG GTC ACA G
VCP	F	GAG GAA TCC TGC TTT ACG GAC C
	R	GGC TTT ACG AAG GTT GCT CTC AG
GAPDH	F	CTC AAG ATC ATC AGC AAT GCC T
	R	AAG TTG TCA TGG ATG ACC TTG G

### Western blot

Extracted proteins were subjected to SDS-PAGE, and the separated proteins were transferred onto polyvinylidene difluoride (PVDF) membranes as previously described (Wu et al., 2021). The membranes were probed with anti-HLA-DRA (Cat #: 97971S, Cell Signaling, Danvers, MA) and anti-β-actin (Cat #: A1978, Sigma-Aldrich, Saint Louis, MO) antibodies, and signals were detected using a LI-COR imaging system (LICORbio, Lincoln, NE).

### Statistical analysis

The experimental results were analyzed using GraphPad Prism 5 software. An unpaired two-tailed *T*-test was used for the comparison of two independent groups. A *p*-value < 0.05 was considered to indicate a statistically significant difference. Single and two asterisks represent a *p*-value of <0.05 and <0.01, respectively. Means ± standard deviation (SD) were shown.

## Results

### Human iPSC-derived iMG

**FIGURE 1:**
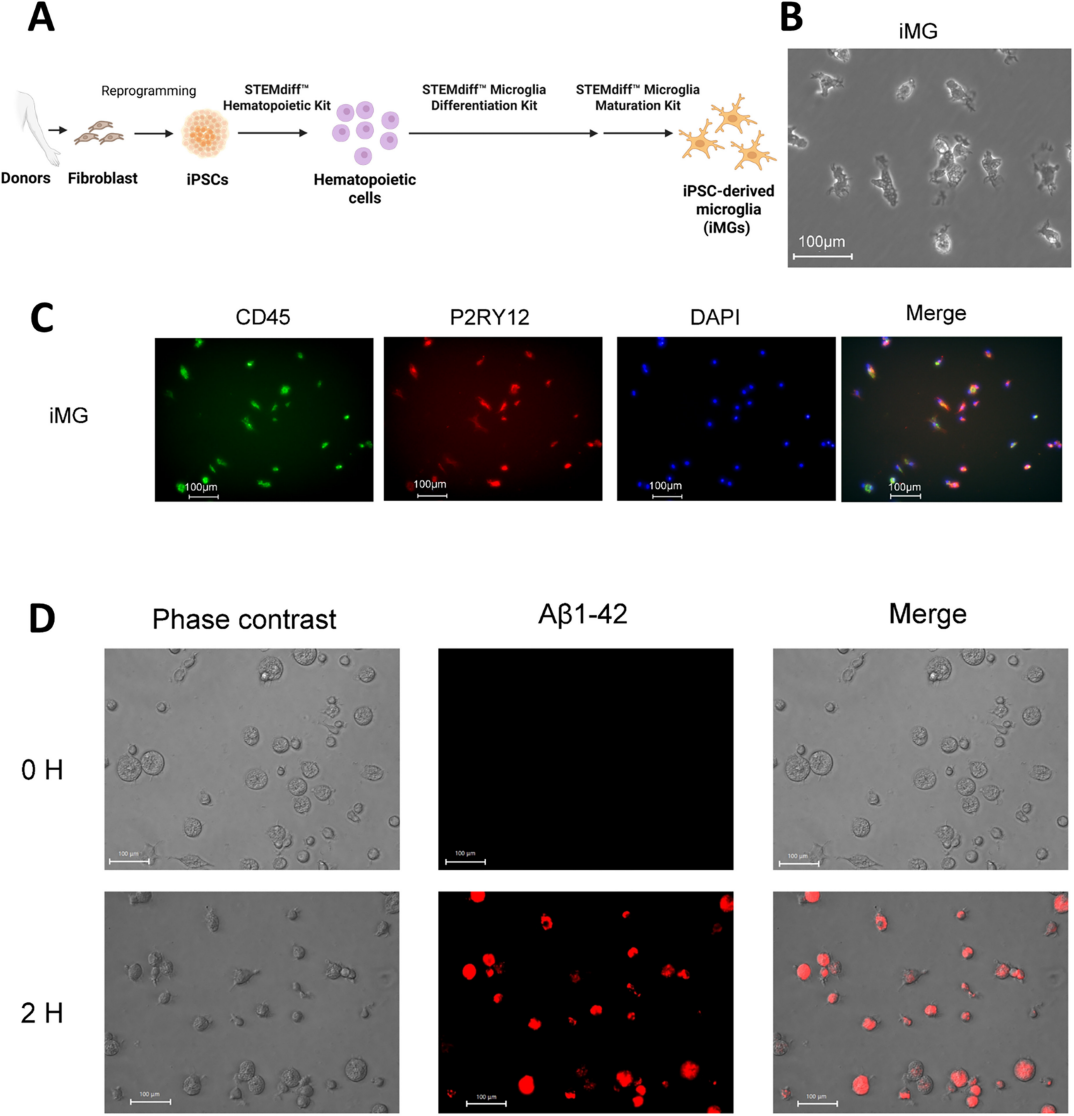
Differentiation of iPSCs-derived microglia (iMG) . **(A)** Schematic workflow of iMG differention. (**B**) Representative phase-contrast image of iMG after 6 days of maturation. **(C)** Immunofluorescence staining showing expression of microglial markers CD45 (green) and P2RY12 (red) after 6 days of maturation. (**D**) Phagocytic activity of iMG. Cells were exposed to Fluor 555-labeled fibrillar Aβ1-42 (15 μg/ml) for 0 or 2 h, followed by fluorescence microscopy visualization of Aβ1-42 uptake. Representative images were shown.

As described, we differentiated iPSCs into HPCs and subsequently into induced microglia (iMG) using StemCell Technologies kits, following the manufacturer’s protocol. A schematic of the differentiation workflow is shown in Figure 1A, and the morphology of iMG is depicted in Figure 1B. P2RY12, a marker selectively expressed by homeostatic microglia, in combination with CD45 expression (P2RY12^+^ CD45^+^), is often used to distinguish resident microglia from peripherally derived myeloid cells (Douvaras et al., 2017; Ritzel et al., 2015; Zhu et al., 2017; Zrzavy et al., 2017). As shown in Figure 1C, these iMGs were positive for CD45 and P2RY12, consistent with a resting microglial phenotype (Haynes et al., 2006; Sedgwick et al., 1991) .

The phagocytic activity of microglia is essential for maintaining brain health and homeostasis by clearing harmful substances, such as amyloid-β (Aβ) deposits. To assess the phagocytic function of iMG, cells were incubated with fibrillar HiLyte Fluor 555-labeled Aβ1-42 for 2 h at 37 °C. As shown in the Figure 1D, internalized fluorescent Aβ was detected in iMG after 2 h, whereas no signal was observed at 0 h, indicating that the iMG were phagocytically functional.

We also included Vero cells, which are derived from the kidney epithelial cells of the African green monkey and are incapable of phagocytosis, to exclude possible passive transport of Aβ1-42 of iMG, and exposed them to fibrillar HiLyte Fluor 555-labeled Aβ1-42 in a similar manner. No internalized fluorescent Aβ was detected, supporting cell-specific phagocytosis (Supplementary Figure 1).

### Sporadic AD-impacted genes

**FIGURE 2:**
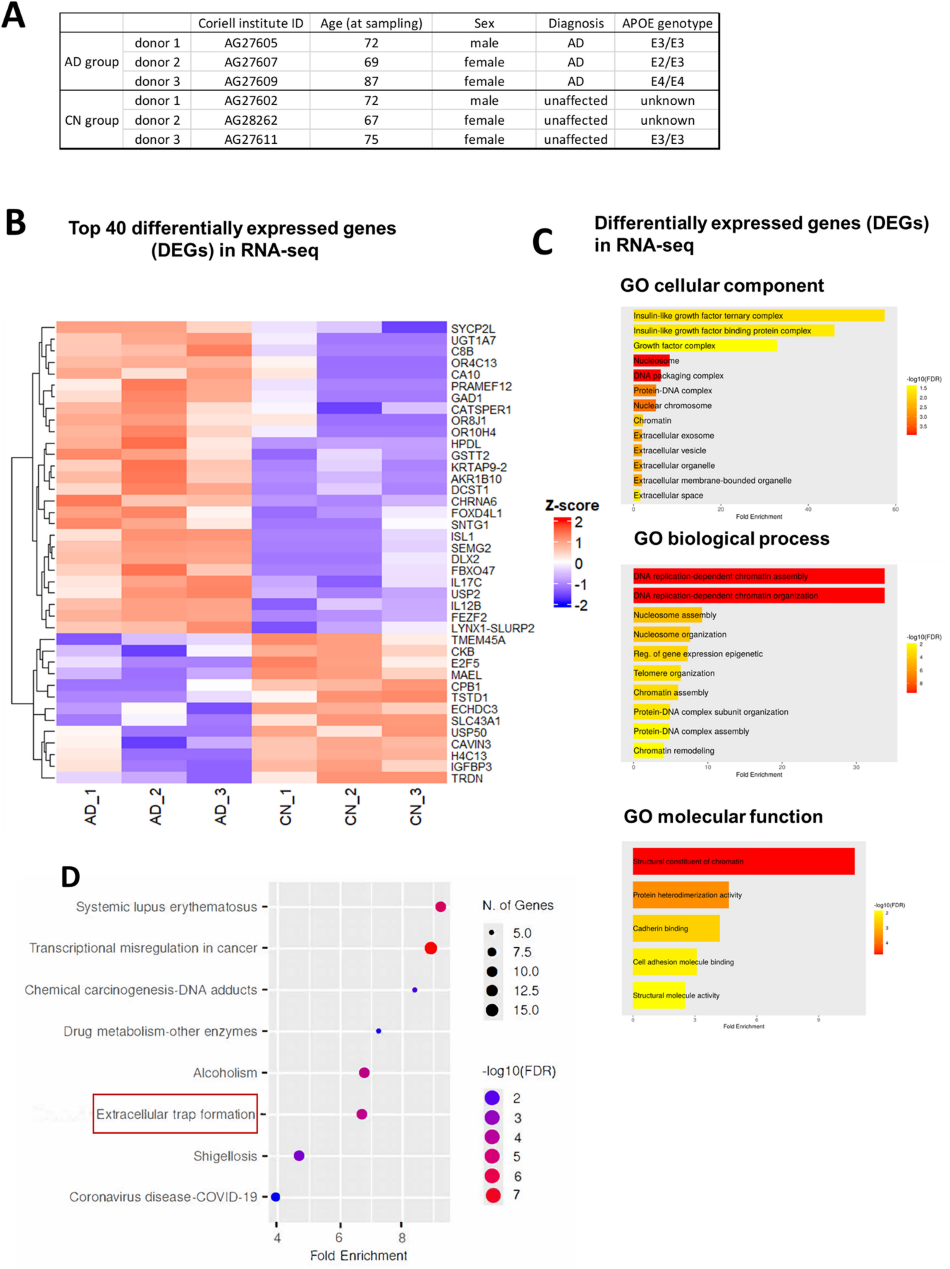
Altered mRNA expression profile in induced pluripotent stem cell (iPSC)-derived microglia (iMGs) from sporadic Alzheimer’s disease (AD) compared with cognitively normal (CN). **(A)** Donor information of induced pluripotent stem cells (iPSCs). **(B)** Heatmap of differentially expressed genes (DEGs) with unsupervised clustering based on Pearson correlation. **(C)** Gene Ontology (GO) enrichment analysis of the DEGs. **(D)** Kyoto Encyclopedia of Genes and Genomes (KEGG) analysis of the DEGs.

To investigate differentially expressed genes (DEGs) in iMGs between LOAD and cognitively normal (CN) individuals, we collected iPSCs from three sporadic AD patients and three age- and sex-matched controls and differentiated them into iMGs. Donor information is provided in the Figure 2A. Total RNA was isolated from the iMGs and subjected to RNA sequencing.

Using a threshold of | log_2_ fold change| > 0.8 and *p*-value < 0.05, we identified 195 differentially expressed genes (DEGs) between LOAD and CN (listed in Table 2). Of these, 95 were downregulated in AD, and 100 were upregulated. A heatmap of the top 40 DEGs, based on the fold change, is shown in the Figure 2B.

**TABLE 2:** Late-onset AD (LOAD)-altered genes in iPSC-derived microglia (iMG).

Gene name	BaseMean	Log_2_ fold change (FC)	*P*-value	CN baseMean	AD baseMean
ABCB1	8.44	2.00	0.003	3.38	13.51
ABCG1	363.52	−0.86	0.046	468.73	258.31
ACD	30.16	−1.13	0.030	41.41	18.91
ACVRL1	26.01	0.82	0.028	18.77	33.24
ADGRE3	60.85	−2.12	0.047	98.98	22.72
AKR1B10	4.58	5.57	0.047	0.19	8.96
AMIGO2	49.57	1.10	0.010	31.48	67.66
ANGPTL2	36.01	1.66	0.014	17.31	54.71
ANKAR	11.89	−1.55	0.022	17.71	6.06
ARHGAP32	61.31	1.07	0.018	39.65	82.98
ARHGAP8	4.19	−1.69	0.043	6.39	1.98
ARMCX2	97.29	−1.06	0.043	131.51	63.08
ARV1	87.74	−0.83	0.038	112.37	63.11
BBOF1	13.78	−1.07	0.014	18.66	8.91
BCL2A1	157.12	1.51	0.026	81.54	232.71
C8B	2.89	4.05	0.024	0.33	5.46
CA10	2.01	2.35	0.040	0.66	3.36
CAMK2N2	4.03	2.09	0.012	1.53	6.52
CATSPER1	11.93	2.81	0.048	2.97	20.89
CAVIN3	10.04	−2.39	0.010	16.85	3.22
CCDC28B	23.63	1.08	0.034	15.14	32.12
CD302	445.83	−0.81	0.038	568.48	323.18
CHRNA6	13.60	2.46	0.022	4.18	23.02
CKB	147.67	−2.65	0.014	254.68	40.67
CLEC4F	11.36	−1.66	0.026	17.25	5.46
CNGB1	12.67	2.30	0.033	4.28	21.06
CNKSR2	4.21	1.39	0.022	2.32	6.09
COMT	191.23	−1.42	0.024	278.63	103.83
CPB1	1.37	−2.80	0.010	2.40	0.34
CPNE2	153.06	−1.31	0.011	218.06	88.06
CREB3L4	23.94	−1.28	0.026	33.94	13.94
CROCC	216.33	1.08	0.049	138.72	293.93
CTLA4	4.99	1.30	0.020	2.89	7.10
CYP3A7	1.18	#NUM!	0.018	2.36	0.00
DCST1	1.65	4.04	0.016	0.19	3.10
DERL3	49.01	−0.81	0.003	62.44	35.57
DIO1	8.29	−1.42	0.007	12.08	4.51
DLX2	3.15	3.56	0.002	0.49	5.80
DRAM1	341.12	0.95	0.002	232.64	449.59
E2F5	11.73	−2.61	0.043	20.16	3.31
ECHDC3	26.36	−3.27	0.017	47.77	4.94
EDA	82.29	−1.07	0.042	111.40	53.19
ENPP7	6.38	1.82	0.017	2.82	9.94
EPB41L1	14.39	1.22	0.031	8.66	20.11
EPOP	21.21	1.82	0.021	9.38	33.05
ERICH6	2.76	2.11	0.046	1.04	4.48
ESRP2	21.65	0.89	0.017	15.18	28.13
FAM20A	282.75	1.05	0.040	183.84	381.65
FBXO47	1.50	3.48	0.037	0.25	2.76
FCGRT	1846.55	−0.91	0.028	2411.86	1281.23
FEZF2	3.66	2.35	0.000	1.20	6.12
FGF11	13.60	−1.72	0.021	20.88	6.32
FLVCR2	242.11	0.87	0.017	171.39	312.82
FLYWCH2	46.45	−1.06	0.007	62.75	30.14
FN3K	35.57	−1.49	0.009	52.48	18.67
FOSL1	26.13	1.48	0.035	13.77	38.49
FOXD4L1	7.98	2.52	0.050	2.38	13.59
FPGT-TNNI	6.66	−1.15	0.028	9.19	4.14
GAD1	1.72	3.23	0.023	0.33	3.10
GIMAP5	64.67	−1.39	0.003	93.58	35.75
GLCCI1	271.40	−1.12	0.026	371.51	171.30
GLIS1	3.95	1.75	0.027	1.82	6.09
GNRHR	18.48	0.89	0.022	12.94	24.02
GPR35	210.94	−0.93	0.021	276.71	145.18
GRID1	13.23	1.73	0.043	6.14	20.31
GSTT1	135.36	−1.03	0.038	181.87	88.85
GSTT2	7.37	3.78	0.035	1.00	13.74
GUCY2D	4.77	1.56	0.038	2.41	7.13
H3C	58.44	−1.57	0.041	87.47	29.41
H4-16	121.08	−1.27	0.038	171.21	70.95
H4C13	5.73	−2.64	0.012	9.87	1.58
H4C5	1530.68	−1.15	0.024	2112.39	948.97
HOXA1	2.42	2.29	0.049	0.82	4.02
HPDL	6.72	2.39	0.047	2.16	11.29
HSPA1A	484.01	−0.90	0.043	631.02	337.00
HTRA1	1361.58	−1.72	0.047	2089.03	634.13
IER3	582.90	0.92	0.048	403.55	762.26
IFT140	138.79	0.85	0.004	99.16	178.43
IGF1	931.24	−1.23	0.046	1306.33	556.14
IGFBP3	400.60	−2.45	0.048	676.97	124.24
IL12B	3.21	3.22	0.001	0.62	5.80
IL17C	5.53	3.22	0.037	1.07	10.00
INPP5F	1365.74	−2.11	0.049	2218.59	512.90
ISL1	1.85	3.81	0.017	0.25	3.45
ITGB7	115.36	−2.22	0.017	189.98	40.74
JAG1	200.49	−1.14	0.015	275.92	125.06
KBTBD13	6.77	1.29	0.029	3.93	9.60
KCNH8	13.53	0.91	0.027	9.41	17.64
KCNQ3	1390.79	−1.28	0.005	1968.47	813.10
KRTAP9-2	1.47	3.87	0.032	0.19	2.76
LILRA6	109.90	1.99	0.018	44.22	175.57
LIN28B	180.58	−1.07	0.037	244.66	116.50
LMOD2	2.29	2.19	0.010	0.82	3.76
LOC110384	3.63	2.06	0.049	1.40	5.86
LPIN1	527.43	1.06	0.034	341.46	713.39
LRATD2	266.81	−0.89	0.013	346.31	187.31
LRP8	296.57	1.11	0.003	187.52	405.61
LRRC4	558.77	−1.55	0.007	833.40	284.14
LRRC43	13.07	−1.36	0.046	18.81	7.33
LYNX1-SLU	5.80	2.91	0.019	1.36	10.23
MAEL	15.30	−2.87	0.011	26.92	3.68
1MAFF	181.87	1.20	0.028	110.48	253.25
MAN1A1	483.31	1.07	0.007	312.37	654.24
MARCHF9	86.18	−1.00	0.034	114.86	57.50
MCEE	31.83	−0.95	0.036	41.97	21.69
MEI4	1.82	#NUM!	0.009	3.64	0.00
MELTF	355.48	−2.02	0.012	570.30	140.65
MFSD2A	331.25	1.05	0.047	215.45	447.06
MND1	17.14	1.22	0.027	10.31	23.97
MUC2	23.72	1.51	0.027	12.30	35.14
MYLIP	91.12	−1.24	0.005	128.15	54.08
NAGS	10.72	−1.69	0.047	16.35	5.09
NEO1	131.55	−1.78	0.004	203.79	59.32
NES	37.42	1.55	0.037	19.07	55.76
NINJ2	121.14	−1.27	0.013	171.32	70.96
NR4A2	42.25	1.39	0.020	23.32	61.19
NR4A3	55.04	1.81	0.016	24.39	85.68
OR10H4	1.70	3.22	0.020	0.33	3.07
OR2T8	9.42	−1.88	0.020	14.83	4.02
OR2W3	26.73	−1.39	0.033	38.73	14.74
OR4C13	2.37	2.63	0.007	0.66	4.08
OR8J1	2.03	2.36	0.045	0.66	3.39
OSCAR	231.74	0.84	0.046	165.85	297.64
OSGIN1	38.08	2.09	0.001	14.50	61.66
PAM	91.32	−0.94	0.006	120.14	62.50
PCDHGB2	23.05	−1.26	0.032	32.50	13.59
PCDHGC3	411.07	−1.08	0.028	558.58	263.57
PDCD5	125.20	−0.86	0.037	161.28	89.13
PGAM2	13.83	−1.10	0.010	18.86	8.79
PHETA2	28.78	−1.99	0.006	45.98	11.58
PHLDA1	380.44	1.82	0.022	168.20	592.67
PHLDA2	1.85	−2.28	0.039	3.06	0.63
PLPP3	142.30	2.07	0.008	54.74	229.86
PMFBP1	65.46	1.82	0.011	28.88	102.05
POU5F1	301.59	1.65	0.044	145.86	457.32
POU6F2	9.35	1.97	0.006	3.79	14.91
PRAMEF12	2.25	3.65	0.048	0.33	4.16
PRKCB	765.98	1.47	0.015	405.47	1126.49
PRR7	4.33	1.55	0.044	2.20	6.46
PYHIN1	8.40	1.51	0.046	4.35	12.44
RAB3IL1	565.89	−0.84	0.027	726.62	405.17
RANBP17	4.81	−1.87	0.039	7.55	2.07
RASAL3	257.87	−0.90	0.028	335.71	180.03
RBM41	291.81	0.90	0.004	203.63	379.98
RGS5	3.25	1.41	0.008	1.78	4.71
RIMBP3C	9.46	1.13	0.044	5.94	12.99
RIMS4	9.41	2.16	0.002	3.44	15.37
RNF145	883.80	−0.93	0.045	1158.90	608.70
RPL23	4875.22	−1.01	0.030	6521.57	3228.87
RPL27A	2927.16	−0.88	0.034	3794.39	2059.93
RPLP1	2253.27	−0.82	0.031	2876.96	1629.59
RPS12	1933.17	−1.15	0.041	2666.35	1200.00
RPS19	3719.71	−1.14	0.024	5119.74	2319.69
RSPH10B2	2.80	−2.09	0.021	4.54	1.06
RTN4RL1	78.84	−1.80	0.032	122.43	35.25
RUNX3	2142.65	−1.04	0.048	2882.39	1402.92
SEMA4B	311.35	−1.18	0.049	431.90	190.81
SEMG2	2.01	3.93	0.003	0.25	3.76
SERHL2	8.64	−2.05	0.026	13.92	3.36
SETSIP	5.77	1.29	0.040	3.35	8.19
SFN	2.14	2.07	0.042	0.82	3.45
SH3BGRL2	10.78	−1.48	0.004	15.88	5.69
SIRPB1	673.63	1.09	0.027	430.25	917.01
SIRPD	3.62	1.97	0.001	1.47	5.78
SKOR1	4.63	1.50	0.049	2.42	6.84
SLC25A43	121.91	−0.89	0.047	158.28	85.54
SLC39A11	456.14	0.83	0.006	327.85	584.44
SLC43A1	4.54	−3.54	0.030	8.36	0.72
SLIT3	443.70	1.56	0.014	224.64	662.77
SMARCD3	18.64	0.94	0.042	12.78	24.51
SMPDL3B	32.84	−0.87	0.009	42.41	23.28
SNTG1	2.26	3.03	0.039	0.49	4.02
SNURF	13.55	−1.04	0.023	18.24	8.85
SPATA31D	4.78	2.10	0.016	1.81	7.76
SPATA6L	3.40	1.53	0.028	1.75	5.06
STARD8	277.61	−0.97	0.023	367.68	187.54
SYCP2L	9.41	2.87	0.013	2.27	16.55
TBX6	6.25	−0.85	0.035	8.05	4.45
TJP1	1515.41	−1.11	0.034	2070.29	960.53
TMEM258	98.23	−1.06	0.026	132.79	63.67
TMEM45A	10.68	−3.48	0.050	19.61	1.75
TMSB15B	15.26	1.43	0.037	8.28	22.24
TRDN	160.45	−2.60	0.033	275.47	45.44
TRIP6	70.48	−1.68	0.025	107.43	33.53
TSPYL5	123.80	−2.14	0.005	201.79	45.81
TSTD1	2.87	−3.97	0.045	5.39	0.34
TTC9	20.11	0.87	0.031	14.23	25.98
TUBA4A	8.77	1.37	0.005	4.89	12.64
TYMP	343.74	0.93	0.015	236.51	450.97
UBQLNL	5.97	1.93	0.045	2.48	9.45
UGT1A7	4.44	3.64	0.007	0.66	8.22
USP2	26.15	2.85	0.037	6.37	45.93
USP50	1.05	−2.49	0.033	1.78	0.32
ZFP3	97.86	−2.34	0.000	163.35	32.36
ZNF728	4.31	1.91	0.031	1.82	6.81

To study the expressed iMG genes, which are potentially affected by AD, DEGs were subjected to Gene Ontology (GO) enrichment analysis, which classified them into three main categories: cellular component, biological process, and molecular function (Figure 2C). Kyoto Encyclopedia of Genes and Genomes (KEGG) analysis, another common functional enrichment analysis for RNA-seq data, was used to identify genes in known biological pathways (Figure 2D).

In the cellular component category, significant enrichment was observed for several categories, ranging from nucleosome, with the most significance (lowest -log10(FDR), to extracellular exosome, extracellular vesicle, extracellular organelle, and extracellular membrane-bounded organelle, which have a slightly less significant level. Impacted genes in cell component category are listed Supplementary Table I. Changes in genes involved in extracellular exosome/vesicles/organelle/membrane-bounded organelles demonstrated that AD significantly impacts formation, composition, release, or regulation of extracellular vehicles (EVs), suggesting that enrichment of EV-related genes in AD iMGs may impair EV-mediated intercellular communication, potentially disrupting microglial interactions with surrounding cells and contributing AD pathogenesis, which is consistent to the reports on the importance of microglia EVs in communicating with neurons, astrocytes, and other microglia (Ghosh and Pearse, 2024), and their roles in spreading tau between neurons, modulating inflammation, and in facilitating disease progression (Weng et al., 2022)

In the biological process category, AD-enriched genes were prominently involved in chromatin assembly, organization, and remodeling, the first two showing the most significant difference between CN and AD iMGs. As the brain’s immune cells, the gene expression of microglia is tightly regulated by chromatin structure. Changes in chromatin regions of genes involved in immune/inflammatory responses, as well as lipid metabolism, have been observed in AD (Li et al., 2023; Sun et al., 2023). The chromatin-associated epigenetic alterations often lead to shifts in microglia states, such as pro-inflammatory and lipid-processing states, contributing to AD pathology. A list of AD-impacted genes involved in microglia chromatin functions is listed in Supplementary Table II.

For the molecular function category, AD-impacted genes are listed in Supplementary Table III, with members of the H3 histone family commonly present in pathways involved in chromatin structure and protein binding, further supporting the importance of microglial chromatin in AD. Besides chromatin proteins, some structural proteins, such as tight junction protein 1 (TJP1) and ribosomal proteins, are also enriched, suggesting microglial differences in cellular membranes and cellular structures for protein synthesis between individuals with and without AD.

### Altered protein profile in iMG by sporadic AD

In addition to RNA analysis, the protein samples of iMGs of AD and CN were subject to proteomics for protein analysis. A total of 617 proteins were detected across all six samples and included in the differential abundance analysis. Using the criteria *p*-value < 0.05, 31 proteins were identified as the differentially expressed proteins (DEPs) (Table 3). Among these, 13 proteins were downregulated, with TSPY-Like Protein 2 (TSPYL2) showing the greatest decrease, while 18 proteins were upregulated, with diazepam binding inhibitor (DBI) showing the most significant increase. DBI is also known as the acyl-CoA binding protein (ACBP), a protein crucial for lipid metabolism and neurotransmission. In AD patients, DBI has been reported to be significantly enhanced in serum (Conti et al., 2021).

**TABLE 3:** Late-onset AD (LOAD)-impacted proteins in iPSC-derived microglia (iMG).

Gene ID	log_2_(LFQ intensity)		Log_2_ fold change (FC)	*P*-value
	CN	AD		
TSPYL2	30.24	26.16	−4.08	0.011
HLA-DRA	29.39	26.52	−2.87	0.005
AIF1	27.26	25.46	−1.80	0.004
SLC25A6	27.36	25.62	−1.75	0.018
NANS	26.64	25.11	−1.53	0.036
NPC2	27.19	25.70	−1.49	0.033
NAGK	26.82	25.36	−1.46	0.017
IFI30	26.95	25.54	−1.41	0.027
CTSZ	26.74	25.40	−1.33	0.027
HNRNPF	26.71	25.57	−1.14	0.034
TTYH3	26.29	25.21	−1.08	0.034
PPP2R2A	26.40	25.32	−1.07	0.021
ATP5PB	26.38	25.37	−1.01	0.047
SH3BGRL	25.61	26.82	1.21	0.046
STIP1	25.62	27.10	1.49	0.040
YWHAZ	28.49	30.06	1.57	0.034
VAT1	27.84	29.42	1.58	0.046
ATP6V1A	26.29	27.94	1.65	0.002
ACTN1	27.57	29.25	1.69	0.032
CAP1	28.35	30.05	1.70	0.036
LCP1	28.84	30.57	1.72	0.036
ATP6V1B2	25.88	27.68	1.80	0.013
RPLP2	26.93	28.73	1.80	0.024
IDH1	26.59	28.56	1.97	0.027
KRT1	29.25	31.27	2.02	0.041
APOE	26.47	28.60	2.13	0.032
VCP	25.91	28.22	2.31	0.004
VIM	31.07	33.51	2.44	0.022
PKM	30.70	33.18	2.47	0.024
CAPG	28.57	31.24	2.67	0.012
DBI	27.54	30.32	2.79	0.046

**FIGURE 3:**
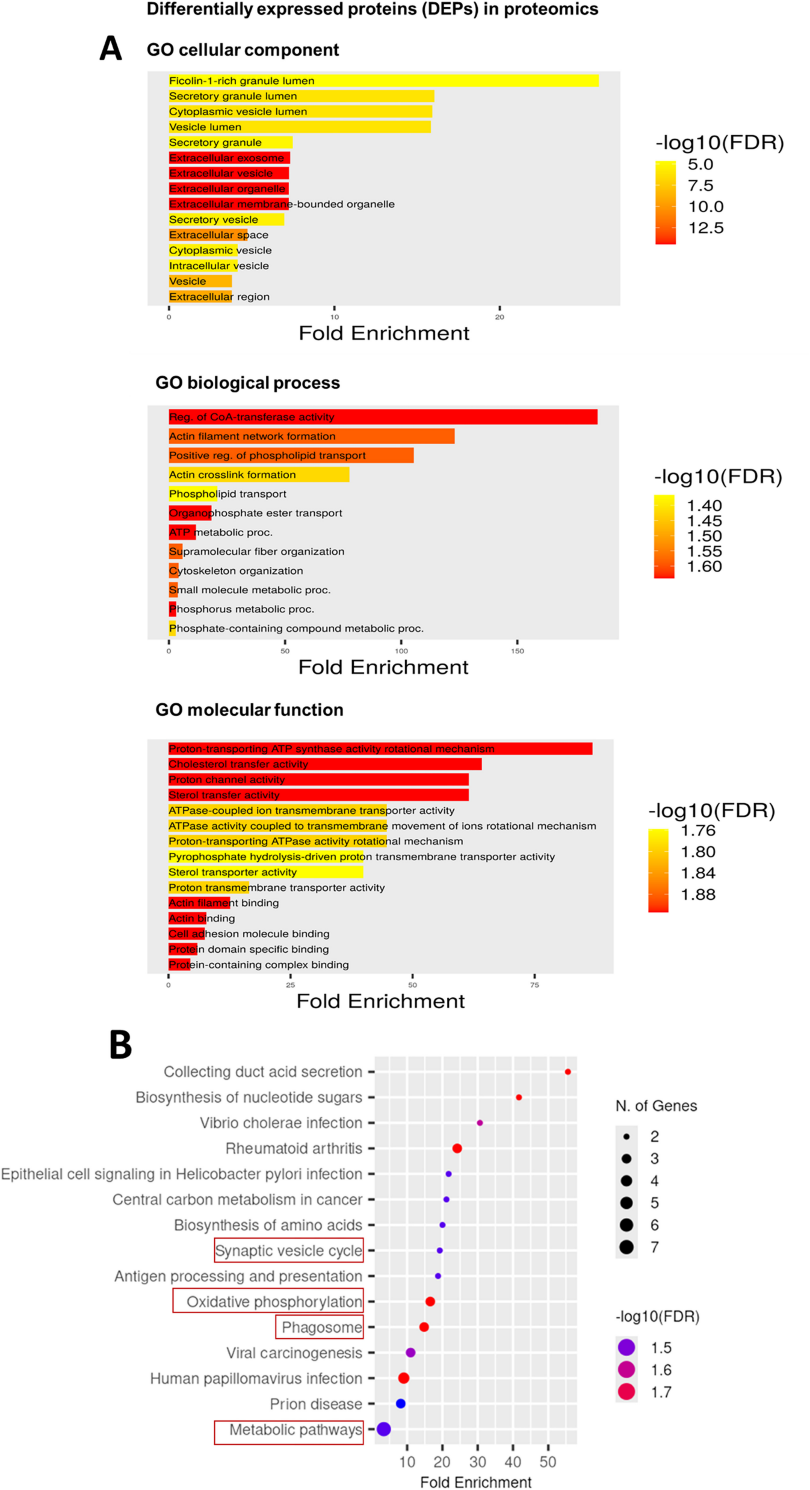
Functional enrichment of differentially expressed proteins (DEPs) in iPSCs-derived microglia (iMGs) from sporadic Alzheimer’s disease (AD). Gene Ontology (GO) enrichment analysis **(A)** and Kyoto Encyclopedia of Genes and Genomes (KEGG) analysis **(B)** of DEPs identified in AD iMGs compared to cognitively normal (CN).

To study the function of these DEPs, GO enrichment analysis was also performed. Significant enrichment was again observed in the categories of cellular component, biological process, and molecular function (Figure 3). At the protein level, extracellular exosomes, extracellular vesicles, extracellular organelles, and extracellular membrane-bounded organelles were the most significantly impacted by AD in iMG, within the category of cell components. In biological process terms, proteins responsible for regulating CoA-transferase activity, phospholipid transport, ATP metabolism, and filament/cytoskeleton organization were all significantly impacted by AD in iMG. Coincidentally, in the category of molecular function, proteins involved in proton-transporting ATP synthase activity, cholesterol transfer activity, and actin binding were also significantly impacted by AD (Figure 3A). The proteins associated with each GO pathway in the categories of cellular component, biological process, and molecular function are listed in Supplementary Tables IV–VI, respectively. KEGG analysis suggested that AD-impacted proteins were essential for metabolic pathways, consistent with the GO analysis, which identified DEPs involved in ATP synthesis and cholesterol transfer. Phagosome and synaptic vesicle cycles revealed by KEGG are also in alignment with genes involved in actin-related activities (Figure 3B).

### Experimental validation of RNA-Seq and proteomics data

**FIGURE 4:**
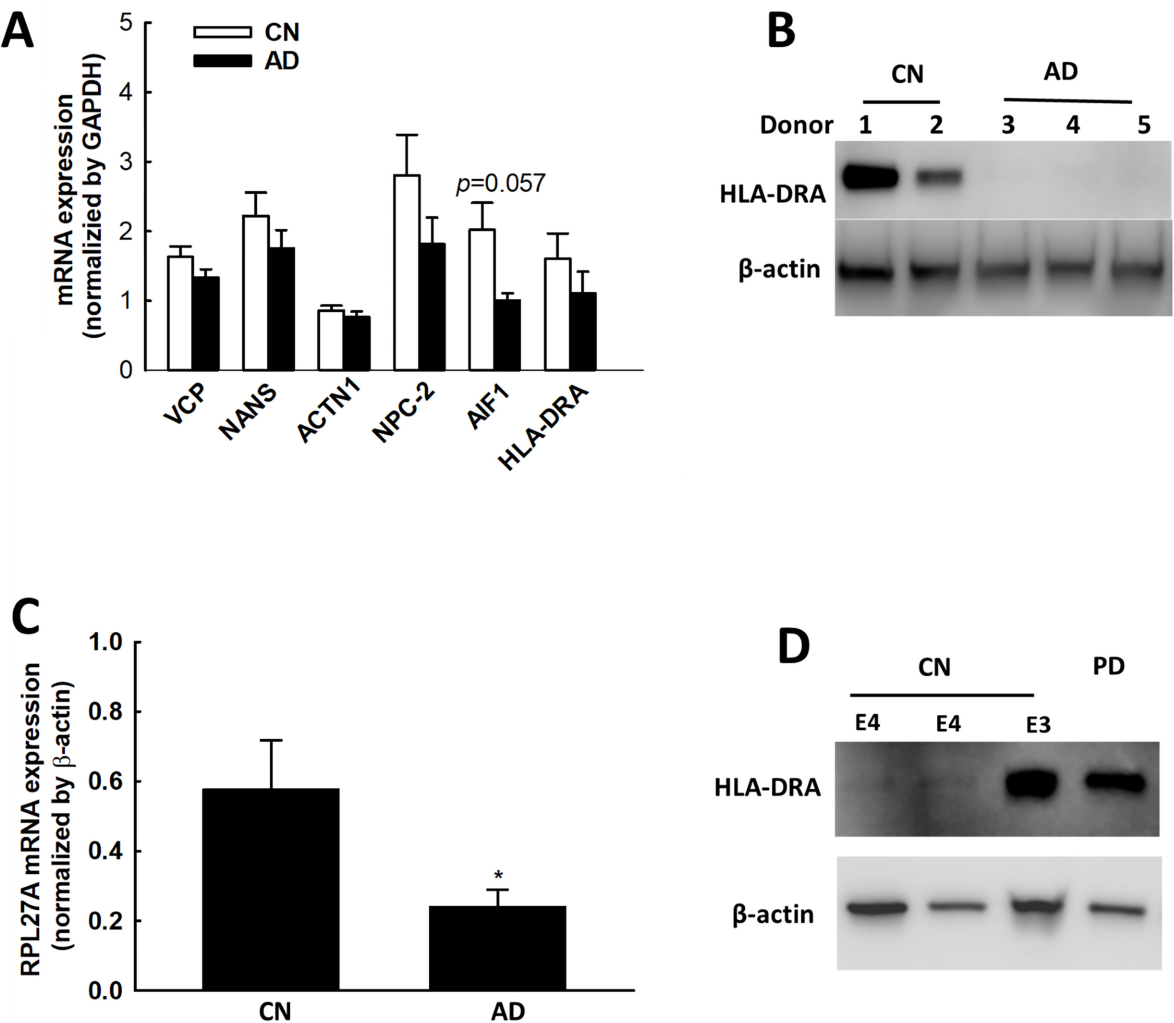
Experimental target validation of iPSCs-derived microglia (iMG) derived from sporadic Alzheimer’s disease (AD) patients and cognitively normal (CN) individuals. **(A)** The mRNA expression of VCP, NANS, ACTN1, NPC-2, AIF1, and HLA-DRA was quantified by qRT-PCR. GAPDH was used as an internal control. In each group, cells were prepared from 3 donors with 2–3 clones/donor. All statistical comparisons were performed using the Mann-Whitney U test. **(B)** Western blot analysis with an human leukocyte antigen DR alpha chain (HLA-DRA) antibody confirmed reduced HLA-DRA expression in sporadic AD. β-actin served as an internal control. **(C)** qRT-PCR was also used to verify the suppression of RPL27A mRNA expression in sporadic AD. A statistical comparison was performed using an unpaired *t*-test; *p < 0.05 relative to the CN group. Data are shown as means ± SE **(D)** HLA-DRA expression was compared between CN iMG with an APOE3 (E3) background and its mutant with APOE4 replacement (E4). The HLA-DRA was also compared between CN E3 and age- and sex-matched PD iMG.

As discussed, RNA-Seq and proteomics were used to identify 13,747 genes with a mean expression level greater than 10 and 617 proteins in iMGs from patients with sporadic AD and healthy subjects. Of these, 195 genes and 31 proteins were detected to be significantly affected by AD. Among the affected proteins in iMG from sporadic AD patients, their corresponding mRNAs were detectable by RNA-seq but did not show significant differences between CN and AD samples, suggesting that the changes in those proteins occurred at the translational level. To test that, we first investigated the mRNA levels of a set of selected proteins listed in Supplementary Table III. Valosin-containing protein (VCP) was present across almost all GO pathways in the cellular component category (Supplementary Table IV), with elevated protein levels in iMG with AD at the most significant level (*P* = 0.004). However, we did not observe changes in mRNA level, revealed by both seq and qRT-PCR (Figure 4A). Similarly, N-acetylneuraminic acid synthase (NANS), alpha-actinin-1 (ACTN1), Niemann-Pick type C-2 (NPC-2), and human leukocyte antigen DR alpha chain (HLA-DRA) did not demonstrate changes in their mRNA expression under the same category.

GO analysis of AD-impacted proteins also revealed pathways under the biological process category (Supplementary Table V), in which VCP and NANS were involved in phosphorus metabolism, and NPC2 was identified as an essential molecule for phospholipid transport. In terms of VCP, it is essential for cell and organ homeostasis and its mutants are linked to the onset and progression of neurodegenerative diseases, such as amyotrophic lateral sclerosis (ALS) and PD (Chu et al., 2023; Clarke et al., 2024). Whether its expression and/or dysfunction contribute to AD is an interesting research topic to be explored. NANS is an enzyme essential for the biosynthesis of N-acetylneuraminic acid, the most common form of sialic acid in humans (Wen et al., 2018). There is no report for NANS in AD pathogenesis. However, since AD is increasingly linked to broader defects in glycosylation and cellular metabolism (Alhasan et al., 2025; Conroy et al., 2021), the association of NANs with AD is worthy of investigation in the near future as well. Interestingly, allograft inflammatory factor 1 (AIF1), a known marker of microglia and neuroinflammation and a regulator of synaptic function (Lituma et al., 2021), was present in five pathways, including actin crosslink formation and fiber organization. AD significantly altered mRNA expression in iMG.

For genes exhibiting comparable mRNA expression between CN and AD iMG in Figure 4A, proteomic analysis revealed AD-associated differences. For validation, we selected HLA-DRA, a known risk factor for late-onset Alzheimer’s disease (LOAD) (Branciamore et al., 2023, 2024). Western blot analysis demonstrated that HLA-DRA protein levels were significantly reduced in AD iMG compared to healthy controls (Figure 4B), supporting that this alteration occurs at the translational level.

Among AD-impacted DEGs, ribosomal protein L27 (RPL27A) showed a significant change (*p* = 0.054) based on RNA-seq data. We reasoned that if a gene showing a borderline-significant change could be validated, other genes with more significant changes would likely be validated as well. The qRT-PCR confirmed a significant change in RPL27A mRNA levels by AD (Figure 4C).

### Changes in HLA-DRA expression in disease conditions

We also investigated whether the suppressed HLA-DRA change observed in Figure 4B is sporadic AD-specific. To achieve this, we developed iMG derived from PD and found that PD did not affect HLA-DRA expression (Figure 4D). APOE4 is a genetic variant (allele) of the apolipoprotein E gene (APOE) that significantly increases the risk of developing AD. Individuals who inherit one or two APOE4 alleles increase their risk, with two copies having the most significant impact (Lin et al., 2018). To investigate whether APOE4 plays a role in HLA-DRA expression in iMG, we obtained iPSC, which were initially derived from healthy donors with the APOE3 allele (CN-E3) but later replaced with the APOE4 allele by CRISPR/Cas9 (CN-E4), from Dr. Tsai LH (Picower Institute for Learning and Memory, Massachusetts Institute of Technology, Cambridge, MA 02139, United States) and developed them to iMG. As shown in Figure 4D, APOE4 suppressed HLA-DRA expression in iMG.

### PSEN1^A246E^ -altered protein expression

**FIGURE 5:**
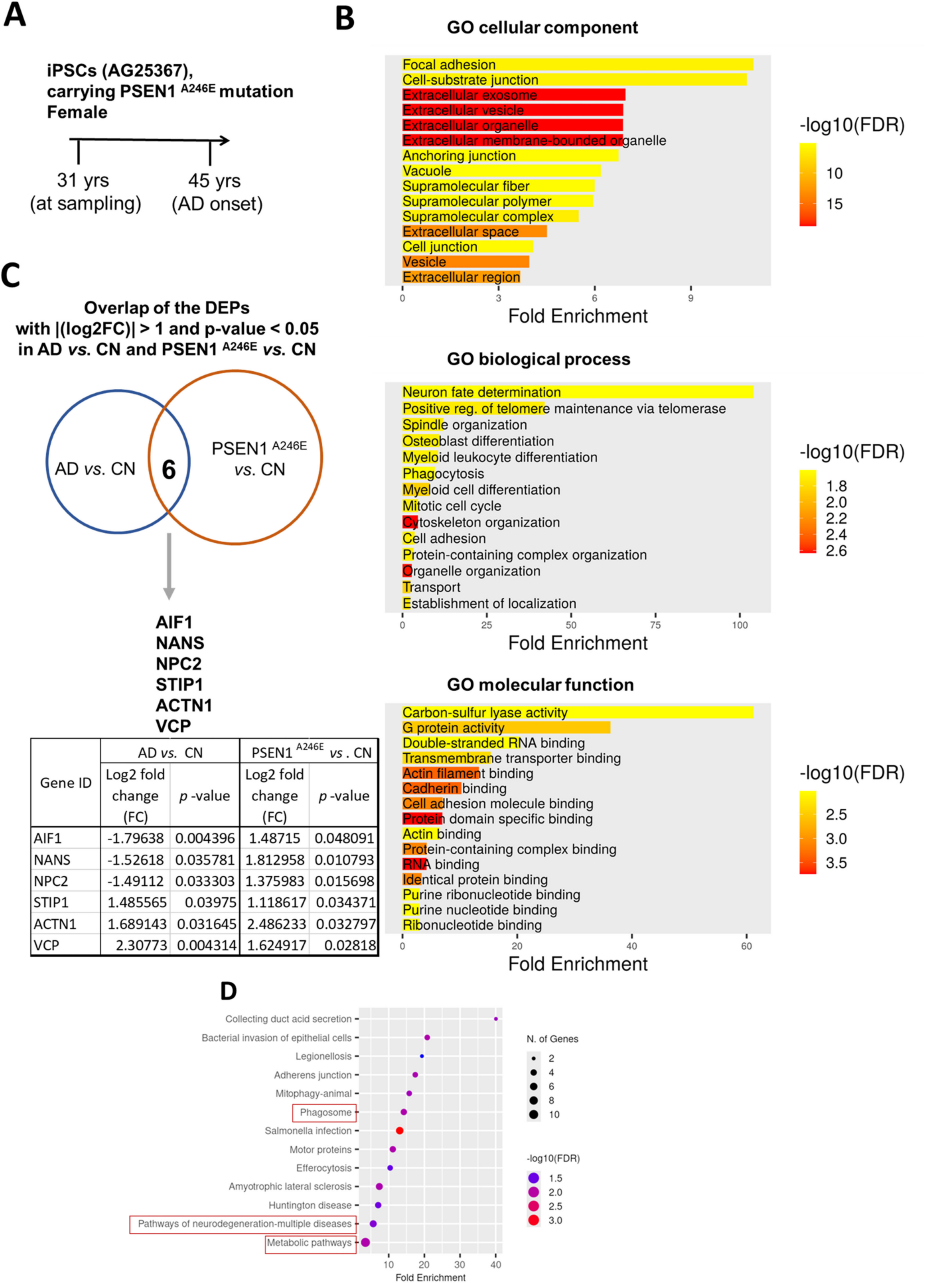
Altered protein expression profile in iPSCs-derived microglia (iMG) of PSEN1^A246E^ compared to age-matched cognitively normal (CN). **(A)** Donor information on induced pluripotent stem cells (iPSCs). **(B)** Gene Ontology (GO) enrichment analysis of differentially expressed proteins (DEPs) between iMG of PSE)N1^A246E^ and CN. **(C)** The list of DEPs that were commonly affected by sporadic Alzheimer’s disease (AD) and early-onset AD with the PSEN1^A246E^ mutation. **(D)** Kyoto Encyclopedia of Genes and Genomes (KEGG) analysis of the DEPs impacted by PSEN1^A246E^ .

Induced pluripotent stem cells line AG25367, carrying the A246E mutation in PSEN1, was derived from a 31-year-old female who was asymptomatic at biopsy but received a diagnosis of early-onset familial AD (EOAD) at age 45 (Figure 5A). Proteomic analysis identified 47 differentially expressed proteins (| log_2_ fold-change| > 1, *P* < 0.05; Table 4), all of which were upregulated in the EOAD model.

**TABLE 4:** Altered protein expression by PSEN1^A246E^ mutation in iPSC-derived microglia (iMG).

Gene ID	log_2_(LFQ intensity)		Log_2_ fold change (FC)	*P*-value
	CN	PSEN1A246E		
HIST1H4A	32.13	33.17	1.04	0.005
KRT18	24.36	27.16	2.80	0.005
HNRNPUL2	25.23	26.31	1.08	0.008
CAPZB	25.76	27.11	1.35	0.008
ATP6V1C1	24.13	26.46	2.33	0.010
NANS	24.59	26.41	1.81	0.011
AK2	25.19	26.56	1.38	0.012
CTNNB1	24.90	25.91	1.00	0.012
RSU1	25.27	26.33	1.05	0.012
CLTC	24.56	27.10	2.54	0.012
RAB7A	24.88	26.52	1.64	0.013
HLA-DRB1	25.24	26.41	1.17	0.014
HNRNPA1	25.09	27.54	2.46	0.015
NPC2	24.86	26.24	1.38	0.016
CA2	24.48	26.02	1.54	0.018
CDC42	25.12	26.55	1.43	0.018
UQCRFS1	24.83	26.17	1.34	0.019
HLA-B/HLA-C	24.47	25.99	1.52	0.020
FLNA	25.12	27.12	2.00	0.021
DCTN2	24.53	26.52	1.99	0.021
HIST2H3A	25.83	27.93	2.10	0.021
ANXA6	25.65	27.62	1.97	0.021
CLIC1	24.58	27.22	2.63	0.022
GNPDA1	25.03	26.30	1.27	0.023
HRNR	24.86	25.96	1.10	0.023
MYH9	24.99	27.99	3.00	0.024
MPP7	24.75	26.58	1.83	0.026
PYCARD	24.80	27.24	2.43	0.026
HSPE1	24.85	27.11	2.26	0.027
VCP	24.62	26.25	1.62	0.028
HMGA1	24.62	26.16	1.54	0.028
PHB2	25.02	26.17	1.15	0.028
TCP1	24.75	26.26	1.50	0.032
TUBB4B	25.43	26.54	1.12	0.033
ACTN1	24.93	27.42	2.49	0.033
STIP1	24.96	26.07	1.12	0.034
SUB1	25.10	26.36	1.26	0.035
FCGBP	25.09	27.24	2.15	0.035
FBP1	25.29	26.41	1.12	0.037
RBMX	24.69	26.12	1.43	0.038
PI4K2A	25.17	26.46	1.29	0.039
RPS4X	24.83	26.25	1.42	0.042
STAB1	25.33	26.44	1.11	0.043
GLO1	26.49	28.79	2.31	0.047
AIF1	24.70	26.19	1.49	0.048
ATP5F1	25.40	26.46	1.06	0.049
GSTM4	25.09	26.19	1.10	0.050

Functional enrichment analysis revealed that EOAD-affected proteins were strongly associated with cellular components, biological processes, and molecular function categories (Figure 5B and Supplementary Tables VII–IX). These enriched categories closely resemble those observed in sporadic AD, highlighting their shared relevance and potential importance in AD pathogenesis.

Among differentially expressed proteins by PSEN1^A246E^ AD and sporadic AD, six proteins were commonly altered in both conditions (Figure 5C). Among these, STIP1, ACTN1, and VCP were upregulated in both PSEN1^A246E^ and sporadic AD. In contrast, AIF1, NANS, and NPC2 were downregulated in sporadic AD but upregulated in PSEN1^A246E^ AD (Figure 5C). Consistent with the KEGG analysis of DEPs for sporadic AD shown in Figure 3B, proteins affected by PSEN1^A246E^ AD were also involved in phagosome and metabolic pathways (Figure 5D).

### Altered sncRNA expression by sporadic AD

Small non-coding RNAs are key regulators of mRNA transcription and protein translation and have been implicated in various neurodegenerative diseases, including AD. In this study, we investigated whether sncRNA expression in iMG is altered by sporadic AD, which accounts for over 90% of all AD cases (Bekris et al., 2010).

**FIGURE 6:**
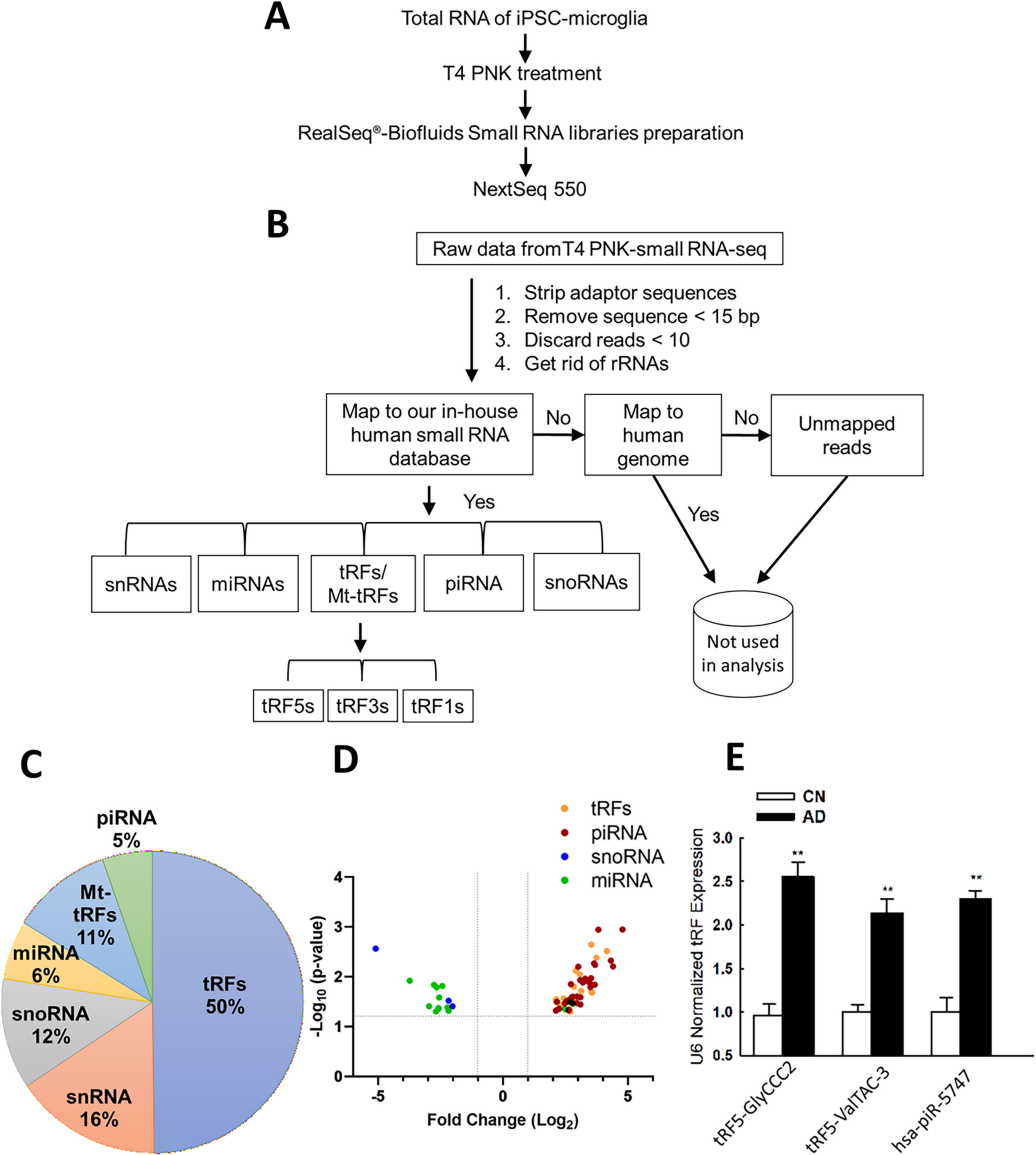
Altered iPSCs-derived microglia (iMG) small non-coding RNAs (sncRNA) expression by sporadic Alzheimer’s disease (AD). **(A)** Brief workflow of T4 PNK-RNA-seq. **(B)** Pipeline for sequencing data analysis. **(C)** The pie chart represented the percentage of raw reads mapping to different sncRNA biotypes. **(D)** The volcano plot showed that sncRNAs were differentially expressed between sporadic AD and cognitively normal (CN). **(E)**. The sncRNA validation for representation tRNA-derived fragments (tRFs) and piRNA. A statistical comparison was performed using an paired *t*-test; **p < 0.01 relative to the CN group. Data are shown as means ± SE.

Our T4 PNK-sncRNA-seq and sequencing analyses, with the overall workflow shown in Figures 6A,B and revealed that tRFs were the most abundant class of sncRNAs across all iMG samples (Figure 6C). As mentioned, the T4-PNK sncRNA seq enabled us to discover several novel AD-impacted tRFs in microglia. Unlike standard small RNA-seq, which relies on 3’-OH and 5’-P for barcode ligation, tRFs with different terminal structures, like 2’,3’-cyclic phosphate at the 3’-ends, cannot be included for sequencing during library construction. T4-PNK treatment provides an effective way to make 3’-ends homogeneous with 3-OH modification and, subsequently, to enable less-biased sequencing (Choi et al., 2020; Wu et al., 2022). Differential expression analysis between AD and CN identified 64 differentially expressed sncRNAs (DEsncRNAs) (| log_2_ fold change| > 1 and *p*-value < 0.05; Table 5). Among these, 33 piRNAs were upregulated, three snoRNAs were downregulated, three miRNAs were upregulated, 11 miRNAs were downregulated, and 16 tRFs were upregulated (Figure 6D and Table 5).

**TABLE 5:** Altered sncRNAs expression in AD iMGs compared to CN.

sncRNA name	BaseMean	Log_2_ fold change (FC)	*p*-value	CN BaseMean	AD BaseMean
hsa-piR-1118	242.93	3.47	0.0132	39.98	445.88
hsa-piR-12264	12.17	3.09	0.0259	2.66	21.68
hsa-piR-12352	69.13	2.22	0.0444	24.48	113.78
hsa-piR-12595	21.74	2.97	0.0248	5.05	38.44
hsa-piR-13787	34.09	3.68	0.0058	4.88	63.30
hsa-piR-15392	24.79	2.64	0.0455	6.98	42.61
hsa-piR-1612	1757.67	3.82	0.0011	233.28	3282.05
hsa-piR-19465	23.20	3.52	0.0107	3.86	42.54
hsa-piR-23041	105.67	3.20	0.0130	20.77	190.58
hsa-piR-23444	578.14	4.32	0.0047	54.82	1101.46
hsa-piR-23446	25.99	3.64	0.0054	3.66	48.32
hsa-piR-24541	537.66	2.67	0.0285	145.78	929.55
hsa-piR-25624	2140.64	3.50	0.0160	347.74	3933.54
hsa-piR-2649	22.88	2.62	0.0469	6.49	39.27
hsa-piR-27429	234.23	2.76	0.0256	60.10	408.36
hsa-piR-27489	17.76	2.54	0.0308	5.55	29.96
hsa-piR-27490	19.39	2.24	0.0438	7.07	31.70
hsa-piR-28345	17.16	2.77	0.0331	4.40	29.91
hsa-piR-30376	10.17	3.34	0.0120	2.01	18.33
hsa-piR-31994	11.63	4.40	0.0062	1.04	22.22
hsa-piR-3411	95.34	2.15	0.0316	35.28	155.40
hsa-piR-3784	16.07	2.96	0.0337	3.71	28.43
hsa-piR-5746	151.03	3.29	0.0109	27.92	274.15
hsa-piR-5747	545.85	2.61	0.0312	153.37	938.32
hsa-piR-7006	17.78	2.47	0.0345	5.44	30.12
SNORD123	40.17	−5.10	0.0027	78.11	2.23
hg38_wgRna_U31	2263.81	−2.18	0.0303	3707.82	819.80
hg38_wgRna_U75	542.92	−2.02	0.0390	871.56	214.27
hsa-let-7b-5p	26.45	2.48	0.0447	8.12	44.78
hsa-miR-143-3p	59.84	−2.68	0.0495	103.14	16.55
hsa-miR-145-5p	62.61	−2.43	0.0153	105.27	19.95
hsa-miR-181a-3p	32.84	−2.56	0.0260	56.42	9.26
hsa-miR-193b-3p	34.55	−2.96	0.0392	60.85	8.25
hsa-miR-224-3p	17.75	−3.74	0.0119	33.26	2.23
hsa-miR-30e-3p	26.68	−2.58	0.0424	45.56	7.79
hsa-miR-361-3p	24.06	−2.65	0.0163	41.60	6.52
hsa-miR-362-5p	18.64	−2.77	0.0145	32.48	4.80
hsa-miR-378a-5p	20.77	−2.18	0.0481	33.91	7.62
hsa-miR-425-3p	31.02	−2.22	0.0411	51.14	10.90
hsa-miR-4286	12.89	2.78	0.0352	3.10	22.68
hsa-miR-451a	38.36	−2.58	0.0437	65.59	11.13
hsa-miR-7977	213.11	2.68	0.0319	57.32	368.90
tRF3-Ala-AGC-1	89.78	2.22	0.0333	31.79	147.78
tRF3-Gln-CTG-1	48.66	3.13	0.0192	9.89	87.44
tRF3-Gln-CTG-2	15.20	4.16	0.0031	1.60	28.80
tRF3-Gln-CTG-5	15.31	2.84	0.0157	3.76	26.86
tRF3-Gln-TTG-3	51.85	3.54	0.0023	8.15	95.54
tRF3-Glu-TTC-11	12.54	3.57	0.0207	2.18	22.89
tRF3-Ser-GCT-5	14.08	2.44	0.0273	4.58	23.58
tRF5-Gly-CCC-2	46.80	2.88	0.0261	11.05	82.54
tRF5-Lys-CTT-10	50.12	2.28	0.0413	17.33	82.91
tRF5-Lys-CTT-16	103.37	2.91	0.0292	24.12	182.61
tRF5-Lys-CTT-6	3865.72	3.54	0.0198	610.38	7121.06
tRF5-Lys-CTT-7	555.67	3.74	0.0042	77.15	1034.19
tRF5-Lys-CTT-5	297.26	2.70	0.0499	79.18	515.33
tRF5-Phe-GAA-1	33.71	2.93	0.0076	8.02	59.40
tRF5-Phe-GAA-4	29.12	3.08	0.0090	6.43	51.81
tRF5-Val-TAC-3	78.68	2.14	0.0282	29.01	128.34

Among the altered tRFs, seven were derived from the 3’ end of tRNAs (tRF3s), and nine were derived from the 5’ end (tRF5s) (Table 5). The upregulated tRF3s originated from tRNA^Ala(AGC)^, tRNA^Gln(CTG/TTG)^, tRNA^Glu(TTC),^ and tRNA^Ser(GCT)^, while the increased tRF5s were derived from tRNA^Gly(CCC)^, tRNA^Lys(CTT)^, tRNA^Phe(GAA)^, and tRNA^Val(TAC)^. These findings suggest that the altered tRF3s and tRF5s originate from distinct tRNAs, potentially indicating different biogenesis or degradation pathways for these tRF subtypes in iMGs derived from sporadic AD patients. We also selected two representative tRFs for the expression validation. As shown in Figure 6E, we confirmed AD-increased expression of tRNA^Gly(CCC)^ and tRNA^Val(TAC)^.

## Discussion

Human iPSCs have become powerful cellular models because they can differentiate into diverse physiologically relevant cell types, particularly those that are otherwise difficult to access. They are widely used for disease modeling, drug discovery, and cell therapy development (Barak et al., 2022; Wang et al., 2021). Many brain cell–derived extracellular vesicles (EVs) have been reported to circulate in the peripheral bloodstream and corresponding EV-based biosensors for precsion diagonostic are being developed for many diseases including AD (Pei et al., 2026). Therefore, molecules identified to be altered in iMG by AD may serve as novel diagnostic biomarkers in the future.

Age is the primary risk factor for sporadic AD. Although reprogramming rejuvenates iPSCs and limits their ability to fully recapitulate the aging complexity of donor cells, iPSC-derived neural cells from patients with sporadic AD recapitulate many disease-associated phenotypes. For instance, iPSC-derived neurons exhibit tau hyperphosphorylation, elevated amyloid levels, mitochondrial dysfunction, and oxidative stress (Ochalek et al., 2017). iPSC-derived astrocytes showed altered calcium signaling and abnormal responses to misfolded protein tau (Brezovakova et al., 2022), while iPSC-derived microglia display impaired phagocytosis (Xu et al., 2019). In this study, we validated the AD-specific HLA-DRA deficiency, using iMG from healthy donors and PD as controls. HLA-DRA deficiency was also associated with APOE4-, not APOE3-, dependent (Figure 4). Therefore, the use of iMGs also provides a controlled and human-relevant system to investigate cell-intrinsic molecular changes while minimizing confounding variables present in primary tissues. Several approaches were proposed to incorporate aging features into studies, such as long-term culture, integrating data from aged primary microglia for validation, and the epigenetic regulation of aging (Jayaraman et al., 2026). Future studies will aim to complement our findings with models that better recapitulate the aging microenvironment to strengthen the translational relevance of our results.

Using iMGs from AD patients and cognitively normal (CN) controls, we examined differential gene (DEG) and protein (DEP) expression profiles. The overlap between DEGs and DEPs was limited, consistent with recent evidence that robust proteomic changes in the AD brain are often not mirrored at the transcriptomic level, underscoring the proteopathic nature of AD (Johnson et al., 2022). Among the DEPs, in addition to HLA-DRA, we identified several novel AD-impacted targets worthy of investigation in the near future. AIF1 was the most significantly altered protein in iMGs from AD samples (*p* = 0.004) and enriched in GO terms related to actin crosslink formation, supramolecular fiber organization, cytoskeleton organization, and phosphorus metabolic processes (Supplementary Table V). AIF1 is a key intracellular signaling molecule involved in phagocytosis, membrane ruffling, and F-actin polymerization. Lituma et al. (2021) developed an AIF1^–^/^–^ murine model in which microglia exhibited reduced ATP-induced motility and ramification, fewer excitatory synaptic connections, and behavioral alterations in adult mice. In future studies, we will investigate whether AD-reduced AIF1 is responsible for impairing human microglial function.

Despite limited overlap at the gene and protein levels, their associated functional GO categories showed substantial convergence. Both DEGs and DEPs were significantly enriched in terms related to the extracellular compartment (e.g., exosomes, vesicles, and organelles) and molecular transport (e.g., ion transport, voltage-gated ion channels, and transmembrane transporter activity), suggesting dysregulated extracellular communication and transport in AD iMGs compared with CN controls (Figures 2C, 3). Multiple altered cargos in neural-derived plasma exosomes or brain tissue-derived extracellular vesicles have been reported in preclinical and diagnosed AD patients, including proteins, RNAs, metabolites, and lipids (Goetzl et al., 2015, 2016; Hernandez et al., 2025; Huang et al., 2024; Jia et al., 2021; Nagaraj et al., 2019; Su et al., 2021, 2022). Abnormal levels of synaptic proteins, inflammatory mediators, growth factors, and lysosomal proteins were detected in exosomes from AD cases compared to controls, suggesting their potential as AD biomarkers (Goetzl et al., 2015, 2016). In addition, oxidative stress in AD brains could alter the cargo composition of exosomes and influence their secretion and intercellular communication (Bir et al., 2024). Consistent with these findings, our results indicate that exosome-related pathways are significantly altered in iMGs derived from AD patients.

Notably, most DEPs in our dataset lacked corresponding mRNA changes, reinforcing that transcriptomics alone may not fully capture the molecular alterations underlying AD pathology. Among DEPs, we validated that AD, not PD, suppressed HLA-DRA expression in iMG (Figure 4), supporting MG-mediated immune dysregulation in AD.

The iPSC line carrying the PSEN1^A246E^ mutation was derived from a presymptomatic familial AD patient with a high genetic risk. DEPs between iMGs derived from PSEN1^A246E^ and age-matched CN controls were enriched in functional categories related to the extracellular compartment (e.g., exosomes, vesicles, and organelles), cytoskeletal organization, transport, and protein binding (e.g., actin filament binding, cell adhesion molecule binding, protein domain–specific binding, and protein complex binding) (Figure 5B). Similar functional categories were also enriched among DEPs by sporadic AD (Figure 3). There were also overlapping DEPs in both PSEN1^A246E^ and sporadic AD iMGs: STIP1, ACTN1, and VCP (Figure 5C). STIP1, which can be secreted by microglia via extracellular vesicles, has been shown to possess neurotrophic properties, and the overexpression has been reported to accelerate amyloid-β deposition in an AD mouse model (Hajj et al., 2013; Lackie et al., 2020; Maciejewski et al., 2016). Elevated levels of STIP1 have also been observed in the brains of AD patients, where it co-localizes with amyloid plaques (Lackie et al., 2020; Ostapchenko et al., 2013). ACTN1 plays a critical role in cytoskeletal organization in microglia and is essential for microglial migration, phagocytosis, and inflammatory responses (Franco-Bocanegra et al., 2019; Uhlemann et al., 2016). VCP is involved in regulating immune activation, lysosomal and autophagic function in microglia, and the clearance of tau in neurons (Clarke et al., 2024; Giong et al., 2024; Saha et al., 2023). Together, these findings suggest that STIP1, ACTN1, and VCP may contribute to both the initiation and progression of AD pathology. In the future, we will study whether these genes were also affected by other reported mutations in PSEN1.

Studies on miRNAs and their roles in AD are relatively extensive, compared to those on other types of sncRNAs. In this study, we found 14 altered miRNAs in iMGs by AD. Many of these, including let-7b-5p, miR-143-3p, miR-181a-3p, miR-193b-3p, miR-30e-3p, and miR-361-3p, have been reported to be involved in AD (Derkow et al., 2018; Ji et al., 2019; Liu et al., 2014; Tuna et al., 2025; Wang et al., 2022; Wu Q. et al., 2019). We initiated our AD research by reanalyzing publicly available GEO DataSets (accession GSE48552), which were not originally designed to study tRFs, to examine changes in tRF expression in the hippocampus. Our analysis revealed significant elevations of tRF5s derived from tRNA^Pro(AGG)^, tRNA^Gly(GCC/CCC2)^, and tRNA^Glu(CTC)^ in the hippocampus of individuals with AD (Wu et al., 2021). Except for tRNAGlyCCC2, increased tRF5s identified in AD-induced microglia (iMG) in our current study are distinct from those observed in the AD hippocampus. Given that microglia represent approximately 10% of the total brain cell population (Salter and Stevens, 2017), tRF alterations specific to microglia in sporadic AD may be masked by dominant tRF signatures from other brain cell types. In future, we will analyze tRF expression profiles in iPSC-derived neurons and astrocytes from both AD and cognitively normal (CN) individuals to further dissect cell–type–specific contributions to tRF dysregulation in AD.

snoRNAs, which typically range from 60 to 300 nucleotides, guide site-specific modifications of ribosomal and spliceosomal RNAs. In our sequencing data, however, snoRNA reads primarily range from 19 to 35 nucleotides, indicating they represent snoRNA-derived small RNA fragments rather than full-length snoRNAs. In AD iMGs, three snoRNA fragments, snoRD123, U31, and U75, were significantly downregulated compared to CN, with snoRD123 showing the largest reduction (∼35-fold). Knowledge of snoRNA-derived small RNA fragments (sdRNAs) is emerging (Wajahat et al., 2021). However, their roles in neurodegenerative diseases are minimal. In terms of piRNAs, they comprised only a small fraction of the total sncRNA population in iMGs (approximately 5%) but were the largest group of differentially expressed sncRNAs (DEsncRNAs) in sporadic AD, consistent with several independent studies reporting changes in piRNA expression in AD brains (Mao et al., 2019; Roy et al., 2017). These findings highlight the potential significance of both piRNAs and snoRNA-derived fragments in AD pathology, particularly in MG-mediated changes, and underscore the need for further investigation into their functional roles.

In summary, we investigated the altered RNA and protein expression profiles in iMGs from AD patients and compared them to those from CN individuals. We identified dysregulation in microglial extracellular communication and transport processes at both presymptomatic and later stages of AD-derived cells. We further analyzed differentially expressed sncRNAs in this cell model, revealing several AD-associated changes. In this study, we validated the AD-specific HLA-DRA deficiency. These results also support iMGs as a useful cellular model for exploring the disease mechanisms underlying AD.

## Data Availability

The original contributions presented in the study are publicly available, and are available in the Supplementary material. This data can be found here: https://www.ncbi.nlm.nih.gov/geo/query/acc.cgi?acc=GSE332551.

## Footnotes

1
https://www.pirnadb.org/
