# Supplementary material for: Disease-associated RNA and protein signatures in iPSC-derived microglia model of Alzheimer’s disease
Source: Front Neurosci. 2026 May 26;20:1799542. doi: 10.3389/fnins.2026.1799542 (PMC13246725; doi:10.3389/fnins.2026.1799542)
Supplement: Supplementary file 1 [file Data_Sheet_1.pdf]

DEG GO: Cellular Component

Enrichment FDR	nGenes	Pathway Genes	Fold Enrichment	Pathway	URL	Genes
0.0168	2	4	57.49	GO:0042567 insulin-like growth factor ternary complex	http://amigo.geneontology.org/amigo/term/GO:0042567	IGF1 IGFBP3
0.0232	2	5	45.99	GO:0016942 insulin-like growth factor binding protein complex	http://amigo.geneontology.org/amigo/term/GO:0016942	IGF1 IGFBP3
0.0445	2	7	32.85	GO:0036454 growth factor complex	http://amigo.geneontology.org/amigo/term/GO:0036454	IGF1 IGFBP3
0.0001	10	139	8.27	GO:0000786 nucleosome	http://amigo.geneontology.org/amigo/term/GO:0000786	H3C12 H3C4 H3C8 H3C6 H3C11 H3C1 H3C7 H3C10 H3C2 H3C3
0.0001	12	222	6.22	GO:0044815 DNA packaging complex	http://amigo.geneontology.org/amigo/term/GO:0044815	SMARCD3 ACD H3C12 H3C4 H3C8 H3C6 H3C11 H3C1 H3C7 H3C10 H3C2 H3C3
0.0010	11	245	5.16	GO:0032993 protein-DNA complex	http://amigo.geneontology.org/amigo/term/GO:0032993	ACD H3C12 H3C4 H3C8 H3C6 H3C11 H3C1 H3C7 H3C10 H3C2 H3C3
0.0006	12	270	5.11	GO:0000228 nuclear chromosome	http://amigo.geneontology.org/amigo/term/GO:0000228	SYCP2L MEI4 H3C12 H3C4 H3C8 H3C6 H3C11 H3C1 H3C7 H3C10 H3C2 H3C3
0.0081	26	1392	2.15	GO:0000785 chromatin	http://amigo.geneontology.org/amigo/term/GO:0000785	SMARCD3 TSPYL5 H3C12 H3C4 H3C8 H3C6 H3C11 H3C1 H3C7 H3C10 H3C2 H3C3 MAEL TBX6 ISL1 RUNX3 POU6F2 DLX2 NR4A3 E2F5 CREB3L4 NR4A2 FOSL1 FOXD4L1 MAFF POU5F1
0.0025	39	2316	1.94	GO:0070062 extracellular exosome	http://amigo.geneontology.org/amigo/term/GO:0070062	CROCC ABCB1 COMT RASAL3 PDCD5 RPS19 FAM20A MAN1A1 SEMG2 RPL23 TUBA4A SMPDL3B GSTT2B ANGPTL2 ITGB7 CPNE2 PAM CNKSR2 MELTF PGAM2 HTRA1 CKB PRKCB OSCAR SFN GRID1 RTN4RL1 H3C12 H3C4 HSPA1A H3C8 H3C6 H3C11 H3C1 GSTT1 H3C7 H3C10 H3C2 H3C3
0.0025	39	2342	1.91	GO:1903561 extracellular vesicle	http://amigo.geneontology.org/amigo/term/GO:1903561	CROCC ABCB1 COMT RASAL3 PDCD5 RPS19 FAM20A MAN1A1 SEMG2 RPL23 TUBA4A SMPDL3B GSTT2B ANGPTL2 ITGB7 CPNE2 PAM CNKSR2 MELTF PGAM2 HTRA1 CKB PRKCB OSCAR SFN GRID1 RTN4RL1 H3C12 H3C4 HSPA1A H3C8 H3C6 H3C11 H3C1 GSTT1 H3C7 H3C10 H3C2 H3C3
0.0025	39	2343	1.91	GO:0043230 extracellular organelle	http://amigo.geneontology.org/amigo/term/GO:0043230	CROCC ABCB1 COMT RASAL3 PDCD5 RPS19 FAM20A MAN1A1 SEMG2 RPL23 TUBA4A SMPDL3B GSTT2B ANGPTL2 ITGB7 CPNE2 PAM CNKSR2 MELTF PGAM2 HTRA1 CKB PRKCB OSCAR SFN GRID1 RTN4RL1 H3C12 H3C4 HSPA1A H3C8 H3C6 H3C11 H3C1 GSTT1 H3C7 H3C10 H3C2 H3C3
0.0025	39	2343	1.91	GO:0065010 extracellular membrane-bounded organelle	http://amigo.geneontology.org/amigo/term/GO:0065010	CROCC ABCB1 COMT RASAL3 PDCD5 RPS19 FAM20A MAN1A1 SEMG2 RPL23 TUBA4A SMPDL3B GSTT2B ANGPTL2 ITGB7 CPNE2 PAM CNKSR2 MELTF PGAM2 HTRA1 CKB PRKCB OSCAR SFN GRID1 RTN4RL1 H3C12 H3C4 HSPA1A H3C8 H3C6 H3C11 H3C1 GSTT1 H3C7 H3C10 H3C2 H3C3
0.0218	49	3577	1.58	GO:0005615 extracellular space	http://amigo.geneontology.org/amigo/term/GO:0005615	CROCC ABCB1 COMT RASAL3 PDCD5 RPS19 FAM20A MAN1A1 SEMG2 RPL23 TUBA4A SMPDL3B GSTT2B ANGPTL2 ITGB7 CPNE2 PAM CNKSR2 MELTF PGAM2 HTRA1 CKB PRKCB OSCAR SFN GRID1 RTN4RL1 H3C12 H3C4 HSPA1A H3C8 H3C6 H3C11 H3C1 GSTT1 H3C7 H3C10 H3C2 H3C3 FCGRT IL12B IGFBP3 CPB1 EDA SEMA4B IGF1 SLIT3 IL17C LRP8

Supplementary Table I : Differentially expressed genes (DEGs) by LOAD in the Cellular Compoent Category
