# Supplementary material for: Disease-associated RNA and protein signatures in iPSC-derived microglia model of Alzheimer’s disease
Source: Front Neurosci. 2026 May 26;20:1799542. doi: 10.3389/fnins.2026.1799542 (PMC13246725; doi:10.3389/fnins.2026.1799542)
Supplement: Supplementary file 2 [file Data_Sheet_2.pdf]

DEG GO: Biological Proccess

Enrichment FDR	nGenes	Pathway Genes	Fold Enrichment	Pathway	URL	Genes
0.0000	10	34	33.82	GO:0006335 DNA replication-dependent chromatin assembly	http://amigo.geneontology.org/amigo/term/GO:0006335	H3C12 H3C4 H3C8 H3C6 H3C11 H3C1 H3C7 H3C10 H3C2 H3C3
0.0000	10	34	33.82	GO:0034723 DNA replication-dependent chromatin organization	http://amigo.geneontology.org/amigo/term/GO:0034723	H3C12 H3C4 H3C8 H3C6 H3C11 H3C1 H3C7 H3C10 H3C2 H3C3
0.0000	11	137	9.23	GO:0006334 nucleosome assembly	http://amigo.geneontology.org/amigo/term/GO:0006334	H3C12 H3C4 H3C8 H3C6 H3C11 H3C1 H3C7 H3C10 H3C2 H3C3 TSPYL5
0.0001	12	181	7.62	GO:0034728 nucleosome organization	http://amigo.geneontology.org/amigo/term/GO:0034728	SMARCD3 H3C12 H3C4 H3C8 H3C6 H3C11 H3C1 H3C7 H3C10 H3C2 H3C3 TSPYL5
0.0002	11	173	7.31	GO:0040029 reg. of gene expression epigenetic	http://amigo.geneontology.org/amigo/term/GO:0040029	H3C12 H3C4 POU5F1 H3C8 H3C6 H3C11 H3C1 H3C7 H3C10 H3C2 H3C3
0.0007	11	199	6.36	GO:0032200 telomere organization	http://amigo.geneontology.org/amigo/term/GO:0032200	ACD H3C12 H3C4 H3C8 H3C6 H3C11 H3C1 H3C7 H3C10 H3C2 H3C3
0.0005	12	230	6.00	GO:0031497 chromatin assembly	http://amigo.geneontology.org/amigo/term/GO:0031497	H3C12 H3C4 POU5F1 H3C8 H3C6 H3C11 H3C1 H3C7 H3C10 H3C2 H3C3 TSPYL5
0.0012	13	305	4.90	GO:0071824 protein-DNA complex subunit organization	http://amigo.geneontology.org/amigo/term/GO:0071824	SMARCD3 RPL23 H3C12 H3C4 H3C8 H3C6 H3C11 H3C1 H3C7 H3C10 H3C2 H3C3 TSPYL5
0.0072	11	261	4.85	GO:0065004 protein-DNA complex assembly	http://amigo.geneontology.org/amigo/term/GO:0065004	H3C12 H3C4 H3C8 H3C6 H3C11 H3C1 H3C7 H3C10 H3C2 H3C3 TSPYL5
0.0142	12	337	4.09	GO:0006338 chromatin remodeling	http://amigo.geneontology.org/amigo/term/GO:0006338	SMARCD3 H3C12 H3C4 H3C8 H3C6 H3C11 H3C1 H3C7 H3C10 H3C2 H3C3 TSPYL5

Supplymentary Table II: Differentially expressed genes (DEGs) by LOAD in the Biological Process Category
