# Supplementary material for: Disease-associated RNA and protein signatures in iPSC-derived microglia model of Alzheimer’s disease
Source: Front Neurosci. 2026 May 26;20:1799542. doi: 10.3389/fnins.2026.1799542 (PMC13246725; doi:10.3389/fnins.2026.1799542)
Supplement: Supplementary file 3 [file Data_Sheet_3.pdf]

DEG GO: Molecular Function

Enrichment FDR	nGenes	Pathway Genes	Fold Enrichment	Pathway	URL	Genes
0.0000	10	107	10.75	GO:0030527 structural constituent of chromatin	http://amigo.geneontology.org/amigo/term/GO:0030527	H3C12 H3C4 H3C8 H3C6 H3C11 H3C1 H3C7 H3C10 H3C2 H3C3
0.0003	15	371	4.65	GO:0046982 protein heterodimerization activity	http://amigo.geneontology.org/amigo/term/GO:0046982	BCL2A1 NR4A2 H3C12 H3C4 H3C8 H3C6 H3C11 H3C1 H3C7 H3C10 H3C2 H3C3 IL12B ABCG1 UGT1A7
0.0026	13	355	4.21	GO:0045296 cadherin binding	http://amigo.geneontology.org/amigo/term/GO:0045296	TJP1 SFN H3C12 H3C4 HSPA1A H3C8 H3C6 H3C11 H3C1 H3C7 H3C10 H3C2 H3C3
0.0088	16	594	3.10	GO:0050839 cell adhesion molecule binding	http://amigo.geneontology.org/amigo/term/GO:0050839	TJP1 SFN H3C12 H3C4 HSPA1A H3C8 H3C6 H3C11 H3C1 H3C7 H3C10 H3C2 H3C3 ITGB7 IGF1 PLPP3
0.0142	20	908	2.53	GO:0005198 structural molecule activity	http://amigo.geneontology.org/amigo/term/GO:0005198	RPS19 RPS12 RPL23 TUBA4A RPLP1 RPL27A CROCC EPB41L1 SNTG1 H3C12 H3C4 H3C8 H3C6 H3C11 H3C1 H3C7 H3C10 H3C2 H3C3 JAG1

Supplementary Table III: Differentially expressed genes (DEGs) by LOAD in the Molecular Function Category
