# Supplementary material for: Disease-associated RNA and protein signatures in iPSC-derived microglia model of Alzheimer’s disease
Source: Front Neurosci. 2026 May 26;20:1799542. doi: 10.3389/fnins.2026.1799542 (PMC13246725; doi:10.3389/fnins.2026.1799542)
Supplement: Supplementary file 4 [file Data_Sheet_4.pdf]

DEPs GO: Cellular Component

Enrichment FDR	nGenes	Pathway Genes	Fold Enrichment	Pathway	URL	Genes
0.0000	5	142	25.99	GO:1904813 ficolin-1-rich granule lumen	http://amigo.geneontology.org/amigo/term/GO:1904813	PKM CTSZ IDH1 VCP KRT1
0.0001	5	192	19.22	GO:0001726 ruffle	http://amigo.geneontology.org/amigo/term/GO:0001726	AIF1 ACTN1 CAPG LCP1 ATP6V1B2
0.0001	5	207	17.83	GO:0005775 vacuolar lumen	http://amigo.geneontology.org/amigo/term/GO:0005775	VAT1 NPC2 CAP1 VCP IFI30
0.0000	8	368	16.05	GO:0034774 secretory granule lumen	http://amigo.geneontology.org/amigo/term/GO:0034774	PKM ACTN1 CTSZ VAT1 NPC2 CAP1 IDH1 VCP
0.0000	8	371	15.92	GO:0060205 cytoplasmic vesicle lumen	http://amigo.geneontology.org/amigo/term/GO:0060205	PKM ACTN1 CTSZ VAT1 NPC2 CAP1 IDH1 VCP
0.0000	8	373	15.83	GO:0031983 vesicle lumen	http://amigo.geneontology.org/amigo/term/GO:0031983	PKM ACTN1 CTSZ VAT1 NPC2 CAP1 IDH1 VCP
0.0000	10	987	7.48	GO:0030141 secretory granule	http://amigo.geneontology.org/amigo/term/GO:0030141	ATP6V1A PKM ACTN1 CTSZ VAT1 NPC2 CAP1 IDH1 VCP KRT1
0.0000	9	901	7.37	GO:0000323 lytic vacuole	http://amigo.geneontology.org/amigo/term/GO:0000323	ATP6V1A ATP6V1B2 CTSZ IFI30 NPC2 HLA-DRA VAT1 CAP1 VCP
0.0000	9	901	7.37	GO:0005764 lysosome	http://amigo.geneontology.org/amigo/term/GO:0005764	ATP6V1A ATP6V1B2 CTSZ IFI30 NPC2 HLA-DRA VAT1 CAP1 VCP
0.0000	23	2316	7.33	GO:0070062 extracellular exosome	http://amigo.geneontology.org/amigo/term/GO:0070062	VIM CAPG PKM ACTN1 NANS CTSZ VAT1 ATP6V1A NPC2 NAGK APOE SH3BGRL CAP1 LCP1 TTYH3 IDH1 ATP6V1B2 DBI YWHAZ VCP KRT1 RPLP2 HLA-DRA
0.0000	23	2342	7.25	GO:1903561 extracellular vesicle	http://amigo.geneontology.org/amigo/term/GO:1903561	VIM CAPG PKM ACTN1 NANS CTSZ VAT1 ATP6V1A NPC2 NAGK APOE SH3BGRL CAP1 LCP1 TTYH3 IDH1 ATP6V1B2 DBI YWHAZ VCP KRT1 RPLP2 HLA-DRA
0.0000	23	2343	7.25	GO:0043230 extracellular organelle	http://amigo.geneontology.org/amigo/term/GO:0043230	VIM CAPG PKM ACTN1 NANS CTSZ VAT1 ATP6V1A NPC2 NAGK APOE SH3BGRL CAP1 LCP1 TTYH3 IDH1 ATP6V1B2 DBI YWHAZ VCP KRT1 RPLP2 HLA-DRA
0.0000	23	2343	7.25	GO:0065010 extracellular membrane-bounded organelle	http://amigo.geneontology.org/amigo/term/GO:0065010	VIM CAPG PKM ACTN1 NANS CTSZ VAT1 ATP6V1A NPC2 NAGK APOE SH3BGRL CAP1 LCP1 TTYH3 IDH1 ATP6V1B2 DBI YWHAZ VCP KRT1 RPLP2 HLA-DRA
0.0000	11	1165	6.97	GO:0099503 secretory vesicle	http://amigo.geneontology.org/amigo/term/GO:0099503	ATP6V1A ATP6V1B2 PKM ACTN1 CTSZ VAT1 NPC2 CAP1 IDH1 VCP KRT1
0.0001	9	1008	6.59	GO:0005773 vacuole	http://amigo.geneontology.org/amigo/term/GO:0005773	ATP6V1A ATP6V1B2 CTSZ IFI30 NPC2 HLA-DRA VAT1 CAP1 VCP
0.0000	23	3577	4.75	GO:0005615 extracellular space	http://amigo.geneontology.org/amigo/term/GO:0005615	VIM CAPG PKM ACTN1 NANS CTSZ VAT1 ATP6V1A NPC2 NAGK APOE SH3BGRL CAP1 LCP1 TTYH3 IDH1 ATP6V1B2 DBI YWHAZ VCP KRT1 RPLP2 HLA-DRA
0.0000	16	2849	4.15	GO:0031410 cytoplasmic vesicle	http://amigo.geneontology.org/amigo/term/GO:0031410	ATP6V1A CTSZ HLA-DRA VIM CAPG ATP6V1B2 YWHAZ PKM ACTN1 VAT1 NPC2 APOE CAP1 IDH1 VCP KRT1
0.0000	16	2851	4.14	GO:0097708 intracellular vesicle	http://amigo.geneontology.org/amigo/term/GO:0097708	ATP6V1A CTSZ HLA-DRA VIM CAPG ATP6V1B2 YWHAZ PKM ACTN1 VAT1 NPC2 APOE CAP1 IDH1 VCP KRT1
0.0000	23	4466	3.80	GO:0031982 vesicle	http://amigo.geneontology.org/amigo/term/GO:0031982	VIM CAPG PKM ACTN1 NANS CTSZ VAT1 ATP6V1A NPC2 NAGK APOE SH3BGRL CAP1 LCP1 TTYH3 IDH1 ATP6V1B2 DBI YWHAZ VCP KRT1 RPLP2 HLA-DRA
0.0000	24	4673	3.79	GO:0005576 extracellular region	http://amigo.geneontology.org/amigo/term/GO:0005576	VIM CAPG PKM ACTN1 NANS CTSZ VAT1 ATP6V1A NPC2 NAGK APOE SH3BGRL CAP1 LCP1 TTYH3 IDH1 ATP6V1B2 DBI YWHAZ VCP KRT1 RPLP2 HLA-DRA IFI30

Supplementary Table IV: Differentially expressed proteins (DEPs) by LOAD in the Cellular Component Category
