# Supplementary material for: Disease-associated RNA and protein signatures in iPSC-derived microglia model of Alzheimer’s disease
Source: Front Neurosci. 2026 May 26;20:1799542. doi: 10.3389/fnins.2026.1799542 (PMC13246725; doi:10.3389/fnins.2026.1799542)
Supplement: Supplementary file 5 [file Data_Sheet_5.pdf]

DEP GO: Biological Proccess

Enrichment FDR	nGenes	athway Gen	Fold Enrichment	Pathway	URL	Genes
0.0229	2	8	184.52	GO:1905918 reg. of CoA-transferase activity	http://amigo.geneontology.org/amigo/term/GO:1905918	APOE DBI
0.0259	2	12	123.02	GO:0051639 actin filament network formation	http://amigo.geneontology.org/amigo/term/GO:0051639	LCP1 ACTN1
0.0259	2	14	105.44	GO:2001140 positive reg. of phospholipid transport	http://amigo.geneontology.org/amigo/term/GO:2001140	APOE DBI
0.0378	2	19	77.69	GO:0051764 actin crosslink formation	http://amigo.geneontology.org/amigo/term/GO:0051764	AIF1 LCP1
0.0431	3	106	20.89	GO:0015914 phospholipid transport	http://amigo.geneontology.org/amigo/term/GO:0015914	APOE DBI NPC2
0.0229	4	161	18.34	GO:0015748 organophosphate ester transport	http://amigo.geneontology.org/amigo/term/GO:0015748	SLC25A6 APOE DBI NPC2
0.0229	5	318	11.61	GO:0046034 ATP metabolic proc.	http://amigo.geneontology.org/amigo/term/GO:0046034	PKM ATP5PB ATP6V1A ATP6V1B2 VCP
0.0259	7	883	5.85	GO:0097435 supramolecular fiber organization	http://amigo.geneontology.org/amigo/term/GO:0097435	VIM CAPG LCP1 KRT1 AIF1 APOE ACTN1
0.0259	9	1594	4.17	GO:0007010 cytoskeleton organization	http://amigo.geneontology.org/amigo/term/GO:0007010	VIM CAPG ACTN1 LCP1 VCP KRT1 AIF1 CAP1 APOE
0.0259	10	1936	3.81	GO:0044281 small molecule metabolic proc.	http://amigo.geneontology.org/amigo/term/GO:0044281	PKM ATP5PB APOE IDH1 DBI ATP6V1A NPC2 NAGK VCP NANS
0.0229	13	3030	3.17	GO:0006793 phosphorus metabolic proc.	http://amigo.geneontology.org/amigo/term/GO:0006793	PKM ATP5PB PPP2R2A APOE DBI TSPYL2 ATP6V1A NAGK IDH1 YWHAZ AIF1 VCP NANS
0.0378	12	3005	2.95	GO:0006796 phosphate-containing compound metabolic proc.	http://amigo.geneontology.org/amigo/term/GO:0006796	PKM ATP5PB PPP2R2A APOE DBI TSPYL2 ATP6V1A NAGK IDH1 YWHAZ AIF1 VCP

Supplementary Table V: Differentially expressed proteins (DEPs) by LOAD in the Biological Process Category
