# Supplementary material for: Disease-associated RNA and protein signatures in iPSC-derived microglia model of Alzheimer’s disease
Source: Front Neurosci. 2026 May 26;20:1799542. doi: 10.3389/fnins.2026.1799542 (PMC13246725; doi:10.3389/fnins.2026.1799542)
Supplement: Supplementary file 6 [file Data_Sheet_6.pdf]

DEP GO: Molecular Function

Enrichment FDR	nGenes	Pathway Genes	Fold Enrichment	Pathway	URL	Genes
0.0122	2	17	86.83	GO:0046933 proton-transporting ATP synthase activity rotational mechanism	http://amigo.geneontology.org/amigo/term/GO:0046933	ATP5PB ATP6V1A
0.0122	2	23	64.18	GO:0120020 cholesterol transfer activity	http://amigo.geneontology.org/amigo/term/GO:0120020	NPC2 APOE
0.0122	2	24	61.51	GO:0015252 proton channel activity	http://amigo.geneontology.org/amigo/term/GO:0015252	ATP5PB ATP6V1A
0.0122	2	24	61.51	GO:0120015 sterol transfer activity	http://amigo.geneontology.org/amigo/term/GO:0120015	NPC2 APOE
0.0160	2	33	44.73	GO:0042625 ATPase-coupled ion transmembrane transporter activity	http://amigo.geneontology.org/amigo/term/GO:0042625	ATP6V1A ATP6V1B2
0.0160	2	33	44.73	GO:0044769 ATPase activity coupled to transmembrane movement of ions rotational mechanism	http://amigo.geneontology.org/amigo/term/GO:0044769	ATP6V1A ATP6V1B2
0.0160	2	33	44.73	GO:0046961 proton-transporting ATPase activity rotational mechanism	http://amigo.geneontology.org/amigo/term/GO:0046961	ATP6V1A ATP6V1B2
0.0175	2	37	39.90	GO:0009678 pyrophosphate hydrolysis-driven proton transmembrane transporter activity	http://amigo.geneontology.org/amigo/term/GO:0009678	ATP6V1A ATP6V1B2
0.0175	2	37	39.90	GO:0015248 sterol transporter activity	http://amigo.geneontology.org/amigo/term/GO:0015248	NPC2 APOE
0.0265	2	50	29.52	GO:0120013 lipid transfer activity	http://amigo.geneontology.org/amigo/term/GO:0120013	NPC2 APOE
0.0387	2	64	23.07	GO:0019829 ATPase-coupled cation transmembrane transporter activity	http://amigo.geneontology.org/amigo/term/GO:0019829	ATP6V1A ATP6V1B2
0.0160	3	134	16.52	GO:0015078 proton transmembrane transporter activity	http://amigo.geneontology.org/amigo/term/GO:0015078	ATP6V1A ATP5PB ATP6V1B2
0.0122	4	234	12.62	GO:0051015 actin filament binding	http://amigo.geneontology.org/amigo/term/GO:0051015	CAPG ACTN1 LCP1 AIF1
0.0183	4	355	8.32	GO:0045296 cadherin binding	http://amigo.geneontology.org/amigo/term/GO:0045296	CAPG PKM IDH1 YWHAZ
0.0122	5	476	7.75	GO:0003779 actin binding	http://amigo.geneontology.org/amigo/term/GO:0003779	CAPG ACTN1 CAP1 LCP1 AIF1
0.0122	6	594	7.46	GO:0050839 cell adhesion molecule binding	http://amigo.geneontology.org/amigo/term/GO:0050839	CAPG PKM IDH1 YWHAZ ACTN1 LCP1
0.0122	6	748	5.92	GO:0019904 protein domain specific binding	http://amigo.geneontology.org/amigo/term/GO:0019904	ACTN1 SH3BGRL YWHAZ VIM CAPG VCP
0.0122	9	1477	4.50	GO:0044877 protein-containing complex binding	http://amigo.geneontology.org/amigo/term/GO:0044877	PKM HLA-DRA CAPG ACTN1 LCP1 AIF1 APOE VCP VIM
0.0308	6	1053	4.21	GO:0008092 cytoskeletal protein binding	http://amigo.geneontology.org/amigo/term/GO:0008092	CAPG ACTN1 CAP1 LCP1 AIF1 APOE
0.0243	7	1346	3.84	GO:0005215 transporter activity	http://amigo.geneontology.org/amigo/term/GO:0005215	ATP6V1A TTYH3 SLC25A6 ATP5PB NPC2 APOE ATP6V1B2

Supplementary Table VI: Differentially expressed proteins (DEPs) by LOAD in the Molecular Function Category
