# Supplementary material for: Disease-associated RNA and protein signatures in iPSC-derived microglia model of Alzheimer’s disease
Source: Front Neurosci. 2026 May 26;20:1799542. doi: 10.3389/fnins.2026.1799542 (PMC13246725; doi:10.3389/fnins.2026.1799542)
Supplement: Supplementary file 7 [file Data_Sheet_7.pdf]

DEP GO: Cellular Component

Enrichment FDR	nGenes	Pathway Genes	Fold Enrichment	Pathway	URL	Genes
0.00004	5	105	24.76	GO:0035578 azurophil granule lumen	http://amigo.geneontology.org/amigo/term/GO:0035578	PYCARD NPC2 VCP TUBB4B HRNR
0.00005	5	113	23.01	GO:0005903 brush border	http://amigo.geneontology.org/amigo/term/GO:0005903	ACTN1 CAPZB MYH9 FLNA CLIC1
0.00032	5	170	15.29	GO:0005766 primary lysosome	http://amigo.geneontology.org/amigo/term/GO:0005766	PYCARD NPC2 VCP TUBB4B HRNR
0.00000	10	475	10.95	GO:0005925 focal adhesion	http://amigo.geneontology.org/amigo/term/GO:0005925	CDC42 ACTN1 HMGA1 CLTC CTNNB1 FLNA ANXA6 RPS4X RSU1 MYH9
0.00000	10	484	10.74	GO:0030055 cell-substrate junction	http://amigo.geneontology.org/amigo/term/GO:0030055	CDC42 ACTN1 HMGA1 CLTC CTNNB1 FLNA ANXA6 RPS4X RSU1 MYH9
0.00000	31	2316	6.96	GO:0070062 extracellular exosome	http://amigo.geneontology.org/amigo/term/GO:0070062	AK2 CDC42 ACTN1 RAB7A CAPZB NANS MYH9 CA2 KRT18 SUB1 GNPDA1 HSPE1 NPC2 TCP1 GLO1 HNRNPA1 CLTC RSU1 ATP6V1C1 FBP1 VCP CTNNB1 DCTN2 TUBB4B HLA-DRB1 FLNA ANXA6 HRNR RPS4X CLIC1 RBMX
0.00000	31	2342	6.88	GO:1903561 extracellular vesicle	http://amigo.geneontology.org/amigo/term/GO:1903561	AK2 CDC42 ACTN1 RAB7A CAPZB NANS MYH9 CA2 KRT18 SUB1 GNPDA1 HSPE1 NPC2 TCP1 GLO1 HNRNPA1 CLTC RSU1 ATP6V1C1 FBP1 VCP CTNNB1 DCTN2 TUBB4B HLA-DRB1 FLNA ANXA6 HRNR RPS4X CLIC1 RBMX
0.00000	31	2343	6.88	GO:0043230 extracellular organelle	http://amigo.geneontology.org/amigo/term/GO:0043230	AK2 CDC42 ACTN1 RAB7A CAPZB NANS MYH9 CA2 KRT18 SUB1 GNPDA1 HSPE1 NPC2 TCP1 GLO1 HNRNPA1 CLTC RSU1 ATP6V1C1 FBP1 VCP CTNNB1 DCTN2 TUBB4B HLA-DRB1 FLNA ANXA6 HRNR RPS4X CLIC1 RBMX
0.00000	31	2343	6.88	GO:0065010 extracellular membrane-bounded organelle	http://amigo.geneontology.org/amigo/term/GO:0065010	AK2 CDC42 ACTN1 RAB7A CAPZB NANS MYH9 CA2 KRT18 SUB1 GNPDA1 HSPE1 NPC2 TCP1 GLO1 HNRNPA1 CLTC RSU1 ATP6V1C1 FBP1 VCP CTNNB1 DCTN2 TUBB4B HLA-DRB1 FLNA ANXA6 HRNR RPS4X CLIC1 RBMX
0.00000	12	926	6.74	GO:0070161 anchoring junction	http://amigo.geneontology.org/amigo/term/GO:0070161	CDC42 ACTN1 KRT18 HMGA1 CLTC CTNNB1 FLNA ANXA6 RPS4X MPP7 RSU1 MYH9
0.00002	11	901	6.35	GO:0000323 lytic vacuole	http://amigo.geneontology.org/amigo/term/GO:0000323	ATP6V1C1 PI4K2A RAB7A HLA-DRB1 NPC2 CLTC ANXA6 PYCARD VCP TUBB4B HRNR
0.00002	11	901	6.35	GO:0005764 lysosome	http://amigo.geneontology.org/amigo/term/GO:0005764	ATP6V1C1 PI4K2A RAB7A HLA-DRB1 NPC2 CLTC ANXA6 PYCARD VCP TUBB4B HRNR
0.00001	12	1008	6.19	GO:0005773 vacuole	http://amigo.geneontology.org/amigo/term/GO:0005773	ATP6V1C1 PI4K2A RAB7A HLA-DRB1 NPC2 CLTC ANXA6 CDC42 PYCARD VCP TUBB4B HRNR
0.00000	13	1127	6.00	GO:0099512 supramolecular fiber	http://amigo.geneontology.org/amigo/term/GO:0099512	ACTN1 KRT18 TUBB4B AIF1 PYCARD MYH9 TCP1 CLTC CAPZB CTNNB1 DCTN2 FLNA HLA-DRB1
0.00000	13	1136	5.95	GO:0099081 supramolecular polymer	http://amigo.geneontology.org/amigo/term/GO:0099081	ACTN1 KRT18 TUBB4B AIF1 PYCARD MYH9 TCP1 CLTC CAPZB CTNNB1 DCTN2 FLNA HLA-DRB1
0.00000	16	1513	5.50	GO:0099080 supramolecular complex	http://amigo.geneontology.org/amigo/term/GO:0099080	ACTN1 KRT18 TUBB4B AIF1 PYCARD CDC42 MYH9 TCP1 CLTC VCP DCTN2 RPS4X CAPZB CTNNB1 FLNA HLA-DRB1
0.00000	31	3577	4.51	GO:0005615 extracellular space	http://amigo.geneontology.org/amigo/term/GO:0005615	AK2 CDC42 ACTN1 RAB7A CAPZB NANS MYH9 CA2 KRT18 SUB1 GNPDA1 HSPE1 NPC2 TCP1 GLO1 HNRNPA1 CLTC RBMX RSU1 ATP6V1C1 FBP1 VCP CTNNB1 DCTN2 TUBB4B HLA-DRB1 FLNA ANXA6 HRNR RPS4X CLIC1
0.00000	18	2293	4.08	GO:0030054 cell junction	http://amigo.geneontology.org/amigo/term/GO:0030054	CDC42 ACTN1 KRT18 HMGA1 CLTC CTNNB1 FLNA ANXA6 RPS4X MPP7 RSU1 RAB7A CAPZB MYH9 ATP6V1C1 PI4K2A VCP PHB2
0.00000	34	4466	3.96	GO:0031982 vesicle	http://amigo.geneontology.org/amigo/term/GO:0031982	AK2 CDC42 ACTN1 RAB7A CAPZB NANS MYH9 CA2 KRT18 SUB1 GNPDA1 HSPE1 NPC2 TCP1 GLO1 HNRNPA1 CLTC RSU1 ATP6V1C1 FBP1 VCP CTNNB1 DCTN2 TUBB4B HLA-DRB1 FLNA ANXA6 HRNR RPS4X CLIC1 PI4K2A RBMX STAB1 PYCARD
0.00000	33	4673	3.67	GO:0005576 extracellular region	http://amigo.geneontology.org/amigo/term/GO:0005576	AK2 CDC42 ACTN1 RAB7A CAPZB NANS MYH9 CA2 KRT18 SUB1 GNPDA1 HSPE1 NPC2 TCP1 GLO1 HNRNPA1 CLTC RBMX RSU1 ATP6V1C1 FBP1 VCP CTNNB1 DCTN2 TUBB4B HLA-DRB1 FLNA ANXA6 HRNR RPS4X CLIC1 GSTM4 PYCARD

Supplementary Table VII: Differentially expressed proteins (DEPs) by PSEN1^{A246E} in the Cellular Component Category
