# Supplementary material for: Disease-associated RNA and protein signatures in iPSC-derived microglia model of Alzheimer’s disease
Source: Front Neurosci. 2026 May 26;20:1799542. doi: 10.3389/fnins.2026.1799542 (PMC13246725; doi:10.3389/fnins.2026.1799542)
Supplement: Supplementary file 8 [file Data_Sheet_8.pdf]

DEP GO : Biological Proccess

Enrichment FDR	nGenes	Pathway Genes	Fold Enrichment	Pathway	URL	Genes
0.02480	2	10	104.00	GO:0048664 neuron fate determination	http://amigo.geneontology.org/amigo/term/GO:0048664	CDC42 CTNNB1
0.01933	3	37	42.16	GO:0032212 positive reg. of telomere maintenance via telomerase	http://amigo.geneontology.org/amigo/term/GO:0032212	HNRNPA1 CTNNB1 TCP1
0.02498	4	151	13.78	GO:0007052 mitotic spindle organization	http://amigo.geneontology.org/amigo/term/GO:0007052	VCP DCTN2 FLNA CLTC
0.01933	5	215	12.09	GO:0007051 spindle organization	http://amigo.geneontology.org/amigo/term/GO:0007051	VCP DCTN2 FLNA MYH9 CLTC
0.02003	5	239	10.88	GO:0001649 osteoblast differentiation	http://amigo.geneontology.org/amigo/term/GO:0001649	HSPE1 CLTC RBMX CTNNB1 CLIC1
0.02067	5	247	10.53	GO:0002573 myeloid leukocyte differentiation	http://amigo.geneontology.org/amigo/term/GO:0002573	HLA-DRB1 GLO1 CTNNB1 MYH9 CDC42
0.02480	5	272	9.56	GO:0006909 phagocytosis	http://amigo.geneontology.org/amigo/term/GO:0006909	RAB7A MYH9 PYCARD AIF1 CDC42
0.01375	7	450	8.09	GO:0030099 myeloid cell differentiation	http://amigo.geneontology.org/amigo/term/GO:0030099	FLNA HLA-DRB1 GLO1 CTNNB1 MYH9 ACTN1 CDC42
0.02487	7	634	5.74	GO:0006897 endocytosis	http://amigo.geneontology.org/amigo/term/GO:0006897	CDC42 CLTC MYH9 PYCARD AIF1 STAB1 RAB7A
0.02003	9	996	4.70	GO:0000278 mitotic cell cycle	http://amigo.geneontology.org/amigo/term/GO:0000278	CLTC VCP DCTN2 TUBB4B FLNA AIF1 CDC42 CTNNB1 PHB2
0.00234	14	1594	4.57	GO:0007010 cytoskeleton organization	http://amigo.geneontology.org/amigo/term/GO:0007010	ACTN1 CAPZB KRT18 VCP DCTN2 TUBB4B AIF1 CDC42 MYH9 CTNNB1 FLNA PYCARD HLA-DRB1 CLTC
0.01984	12	1729	3.61	GO:0007155 cell adhesion	http://amigo.geneontology.org/amigo/term/GO:0007155	MYH9 FLNA CLIC1 HLA-DRB1 CDC42 AIF1 STAB1 PYCARD KRT18 CTNNB1 ACTN1 RSU1
0.02480	12	1859	3.36	GO:0065003 protein-containing complex assembly	http://amigo.geneontology.org/amigo/term/GO:0065003	CAPZB CLTC HLA-DRB1 PYCARD MPP7 VCP AIF1 RBMX SUB1 UQCRFS1 CDC42 TCP1
0.02003	13	2069	3.27	GO:0043933 protein-containing complex organization	http://amigo.geneontology.org/amigo/term/GO:0043933	CAPZB CLTC VCP HLA-DRB1 PYCARD MPP7 AIF1 RBMX SUB1 UQCRFS1 CDC42 TCP1 HMGA1
0.02480	14	2509	2.90	GO:0051128 reg. of cellular component organization	http://amigo.geneontology.org/amigo/term/GO:0051128	CAPZB CDC42 PYCARD HNRNPA1 MPP7 FBP1 VCP CTNNB1 FLNA MYH9 TCP1 CLTC RAB7A
0.00242	22	4206	2.72	GO:0006996 organelle organization	http://amigo.geneontology.org/amigo/term/GO:0006996	ACTN1 RAB7A CAPZB KRT18 PI4K2A VCP DCTN2 TUBB4B AIF1 PHB2 CDC42 MYH9 PYCARD HNRNPA1 CTNNB1 FLNA HLA-DRB1 SUB1 TCP1 CLTC UQCRFS1 HMGA1
0.01375	22	4823	2.37	GO:0006810 transport	http://amigo.geneontology.org/amigo/term/GO:0006810	CDC42 RAB7A CA2 NPC2 CLTC VCP CLIC1 HNRNPA1 FLNA KRT18 CTNNB1 PHB2 MYH9 PYCARD TCP1 ATP6V1C1 UQCRFS1 DCTN2 ANXA6 AIF1 HLA-DRB1 STAB1
0.01720	22	4987	2.29	GO:0051234 establishment of localization	http://amigo.geneontology.org/amigo/term/GO:0051234	CDC42 RAB7A CA2 NPC2 CLTC VCP CLIC1 HNRNPA1 FLNA KRT18 CTNNB1 PHB2 MYH9 PYCARD TCP1 ATP6V1C1 UQCRFS1 DCTN2 ANXA6 AIF1 HLA-DRB1 STAB1

Supplementary Table VIII: Differentially expressed proteins (DEPs) by PSEN1 A246E in the Biological Process
