# Supplementary figures and images for: Disease-associated RNA and protein signatures in iPSC-derived microglia model of Alzheimer’s disease
Source: Front Neurosci. 2026 May 26;20:1799542. doi: 10.3389/fnins.2026.1799542 (PMC13246725; doi:10.3389/fnins.2026.1799542)

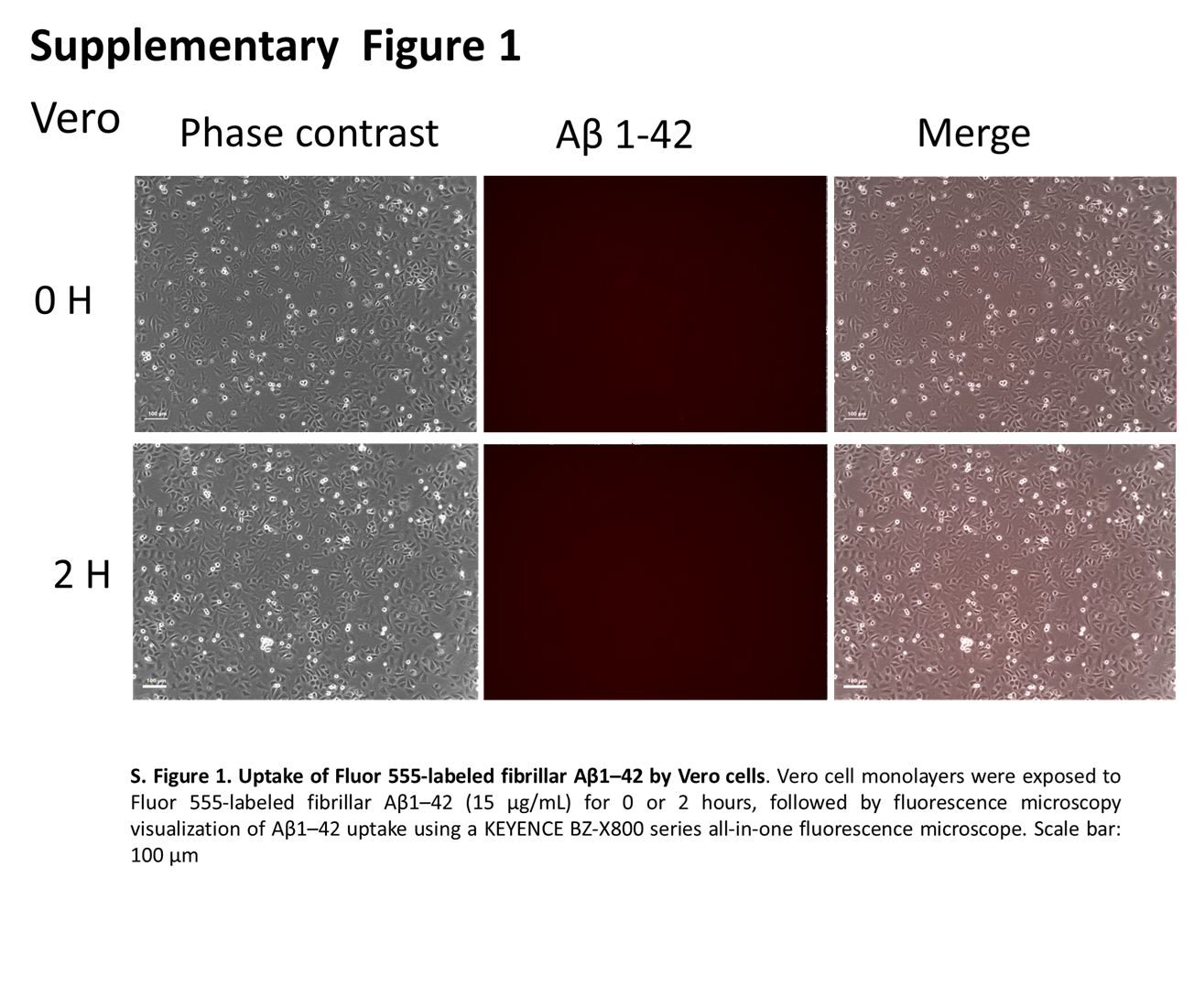

Supplement: Supplementary file 9 [file Image_1.tif]
